# Innovative Driver Monitoring Systems and On-Board-Vehicle Devices in a Smart-Road Scenario Based on the Internet of Vehicle Paradigm: A Literature and Commercial Solutions Overview

**DOI:** 10.3390/s25020562

**Published:** 2025-01-19

**Authors:** Paolo Visconti, Giuseppe Rausa, Carolina Del-Valle-Soto, Ramiro Velázquez, Donato Cafagna, Roberto De Fazio

**Affiliations:** 1 Department of Innovation Engineering, University of Salento, 73100 Lecce, Italy; giuseppe.rausa@unisalento.it (G.R.); donato.cafagna@unisalento.it (D.C.); roberto.defazio@unisalento.it (R.D.F.); 2 Facultad de Ingeniería, Universidad Panamericana, Zapopan 45010, Mexico; cvalle@up.edu.mx; 3 Facultad de Ingeniería, Universidad Panamericana, Aguascalientes 20296, Mexico; rvelazquez@up.edu.mx

**Keywords:** internet of vehicles, intelligent transport system, wearable device, driver monitoring, traffic management, OBD-II, vehicle condition monitoring

## Abstract

In recent years, the growing number of vehicles on the road have exacerbated issues related to safety and traffic congestion. However, the advent of the Internet of Vehicles (IoV) holds the potential to transform mobility, enhance traffic management and safety, and create smarter, more interconnected road networks. This paper addresses key road safety concerns, focusing on driver condition detection, vehicle monitoring, and traffic and road management. Specifically, various models proposed in the literature for monitoring the driver’s health and detecting anomalies, drowsiness, and impairment due to alcohol consumption are illustrated. The paper describes vehicle condition monitoring architectures, including diagnostic solutions for identifying anomalies, malfunctions, and instability while driving on slippery or wet roads. It also covers systems for classifying driving style, as well as tire and emissions monitoring. Moreover, the paper provides a detailed overview of the proposed traffic monitoring and management solutions, along with systems for monitoring road and environmental conditions, including the sensors used and the Machine Learning (ML) algorithms implemented. Finally, this review also presents an overview of innovative commercial solutions, illustrating advanced devices for driver monitoring, vehicle condition assessment, and traffic and road management.

## 1. Introduction

**Figure 1:**
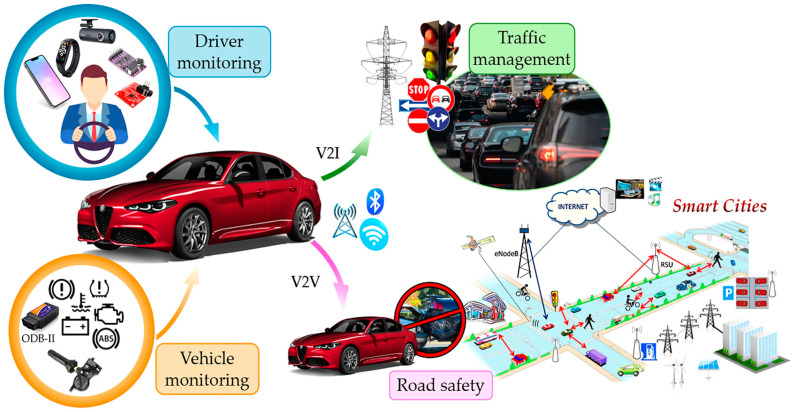
Summary picture of the IoV paradigm in the smart city scenario for road safety and transportation efficiency purposes: driver, vehicle, road, and traffic monitoring systems.

In recent years, growing urbanization and the increasing number of vehicles on the road have introduced new challenges related to transportation safety, efficiency, and sustainability, leading to growing interest in the Internet of Vehicles (IoV) [1]. Within this framework, the monitoring systems of driver, vehicle, and road conditions have become key components of Intelligent Transportation Systems (ITSs) and smart cities [2]. These systems leverage advanced technologies, including sensors, IoT devices, and Artificial Intelligence (AI) to collect, analyze, and utilize real-time data. Their objectives include optimizing traffic flows, enhancing road safety, safeguarding drivers, and reducing environmental impact (Figure 1) [3]. The automotive industry has recently undergone rapid advancements in driver assistance technologies, notably with the development of advanced driver assistance systems (ADASs) [4,5]. These systems represent a crucial step toward future mobility [6]. However, addressing road safety, transportation efficiency, sustainability, and increasingly complex driving conditions requires innovative solutions, particularly to ensure the safety of all road users [7]. In this regard, Driver Monitoring Systems (DMSs) are gaining prominence for their ability to detect health anomalies, drowsiness, intoxication, and unsafe driving behaviors [8]. Recent studies indicate that human errors account for approximately 90% of road accidents, many of which could be prevented with effective monitoring systems [9,10]. These systems rely on both intrusive and non-intrusive sensors to monitor drivers’ biophysical parameters (such as Electrocardiograms (ECGs), photoplethysmography (PPG), Electrodermal Activity (EDA), heart rate (HR), Blood Alcohol Concentration (BAC), respiratory rate (RR), and oxygen saturation (SpO_2_)) and cameras to detect facial cues like yawning, blink rate, and head movements. Using Machine Learning (ML) algorithms and real-time data analysis, these systems enable timely interventions, preventing accidents and saving lives [11,12,13,14,15]. Research has also addressed the recurring tragedy of infants being accidentally left in vehicles. Innovative systems now exist that can detect an infant’s cries and send alert signals through mobile applications, prompting immediate action [16,17]. Investing in such technologies enhances road safety and fosters a more responsible and conscientious driving environment.

In this context, advanced on-board diagnostic (OBD-II) technologies play a pivotal role, not only enhancing vehicle reliability and durability but also directly improving road user safety [18,19]. Many innovative systems utilize data collected through the vehicle’s OBD-II system to monitor its status. Another significant aspect of these technologies is emissions monitoring, which is increasingly important given the tightening of environmental regulations worldwide. Real-time detection of polluting emissions helps minimize environmental impact and optimize resource use [20]. Tire pressure and wear monitoring extend beyond routine maintenance. Proper tire management improves fuel efficiency and reduces the risk of accidents caused by blowouts or loss of control [21]. Similarly, vehicle stability is a fundamental safety factor. Real-time monitoring systems can detect anomalies in driving parameters, preventing potentially hazardous situations [22].

Traffic and road condition monitoring are essential for ensuring urban mobility’s safety, efficiency, and sustainability. Advanced systems incorporating sensors, cameras, and real-time communication technologies collect and analyze data on traffic patterns and infrastructure conditions [23]. This information exchange has given rise to interactive Vehicle-to-Infrastructure (V2I) communication networks, leveraging protocols like Wi-Fi, LoRa (Long Range), 4G/5G, and other technologies to support seamless connectivity and enhanced mobility [24]. Another critical aspect is traffic management, which focuses on optimizing vehicle flow, reducing congestion, and enhancing the driving experience through intelligent planning and innovative solutions such as smart traffic lights and dynamic signaling systems [25]. Additionally, vehicular traffic has been harnessed as a source of energy, enabling the development of systems that harvest, store, and supply energy to power road infrastructure [26]. Monitoring road conditions is equally vital, allowing for the timely detection and resolution of issues like potholes and asphalt deterioration, thereby improving both safety and comfort for road users [27]. Implemented cooperatively, all previous practices enhance urban living standards and support environmental sustainability by fostering smarter, more responsible mobility solutions [28].

Vehicle health monitoring systems are an essential aid to the driver since they provide safety support, such as DMSs used to detect driver anomalies, like anxiety, drowsiness, fatigue, or drunkenness, which are all conditions that significantly reduce attention while driving. DMSs combined with a traffic monitoring and management system create smart frameworks for improving road safety, as by monitoring the vehicles’ flow on the roads they can provide alternative routes in case of emergency, reducing the risk of accidents. Also, the driver’s condition information can be jointly processed and evaluated along with the vehicle parameters to implement smarter DMSs, determining a cumulative driver risk that also takes into account the vehicle conditions (brake wear, tire pressure and wear, etc.). For these reasons, the proposed manuscript explores recent advancements in intelligent driver, vehicle, and traffic monitoring systems to bring out insights and trends for the development of road safety systems in the near future.

This manuscript aims to provide the reader with an overview of IoV solutions in the ITS and smart cities paradigm, analyzing three different macro categories:

➢ Driver monitoring: various on-board applications for driver monitoring are presented, focusing on detecting health anomalies, drowsiness, stress, and impairment due to alcohol consumption. Additionally, the intrusiveness of these applications and their impact on user ergonomics are analyzed (Section 2).
➢ Vehicle condition monitoring: on-board technical solutions for vehicle condition monitoring (i.e., tire pressure and wear, exhaust emissions, and vehicle stability) are examined alongside innovative diagnostic systems and devices for detecting road conditions. Additionally, systems for monitoring driving style and driver behavior are analyzed (Section 3).
➢ Road and environmental monitoring, traffic management: an overview is presented of innovative off-board systems for traffic management, environmental monitoring, and road monitoring, including predictive maintenance solutions. These systems also offer capabilities for detecting road congestion and providing drivers with alternative routes (Section 4).

To enhance the manuscript’s scientific contribution, each section includes a comparative analysis of the reviewed articles, along with summary tables highlighting their key features. Additionally, Section 5 presents innovative commercial solutions developed by international companies for monitoring drivers, vehicles, and traffic conditions.

The structure of this review paper is as follows. Section 1 introduces the topics covered and explains the methodology used for selecting the scientific articles. Section 2 provides an overview of on-board solutions for driver monitoring, organized into three subsections focusing on applications for detecting driver status. Section 3 examines on-board solutions for vehicle condition monitoring, divided into five subsections covering vehicle diagnostics, asphalt monitoring systems, driving style and behavior analysis, tire monitoring, and emissions monitoring. Section 4 explores solutions for traffic monitoring and management, as well as road and environmental condition monitoring. Section 5 reviews innovative commercial applications related to the discussed topics. Finally, Section 6 presents comments and conclusions, emphasizing potential future applications.

### Selection Method of Analyzed Articles Based on PRISMA Methodologies

This section outlines the criteria used for selecting scientific articles, focusing on key aspects such as relevance to the covered topics, publication year, and redundancy with other works. The objective of this review is to provide readers with a comprehensive, state-of-the-art overview of IoV and ITS paradigm applications. The article selection process followed the PRISMA methodology, ensuring the reliability and applicability of the chosen approach [29,30]. The selection process began with evaluating the title of each candidate article to identify keywords related to the topic. Next, the abstract was reviewed to assess its alignment with the issues addressed in this review. If the article appeared relevant, it was thoroughly read and analyzed. Additional sources were consulted to resolve ambiguities for articles with unclear topics. If the content remained unclear after further investigation, the article was excluded.

**Figure 2:**
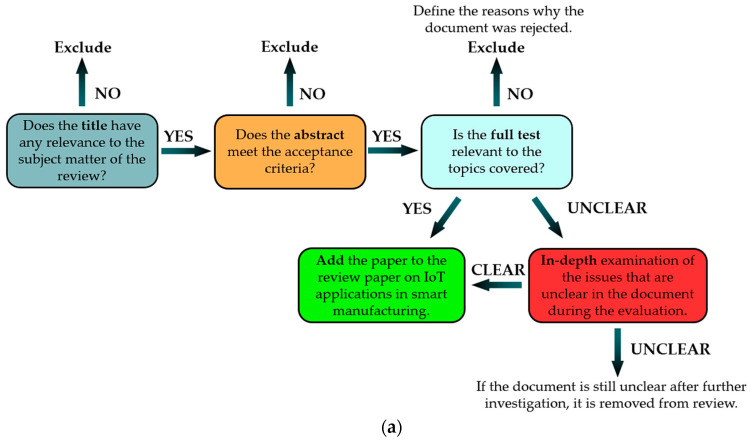

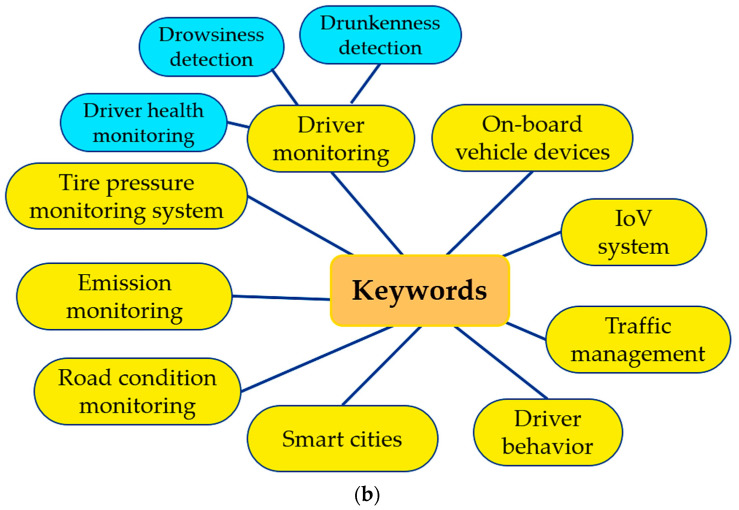
Document selection method: (**a**) description of articles’ selecting method with topics related to the presented review paper, (**b**) main keywords to filter the documents found in the literature.

Figure 2a illustrates the selection process, which included assessing the title’s relevance, the abstract’s affinity, and the manuscript’s scientific value. Figure 2b depicts the main keywords used to filter the literature. This methodology was consistently applied across all topics discussed in this review.

**Figure 3:**
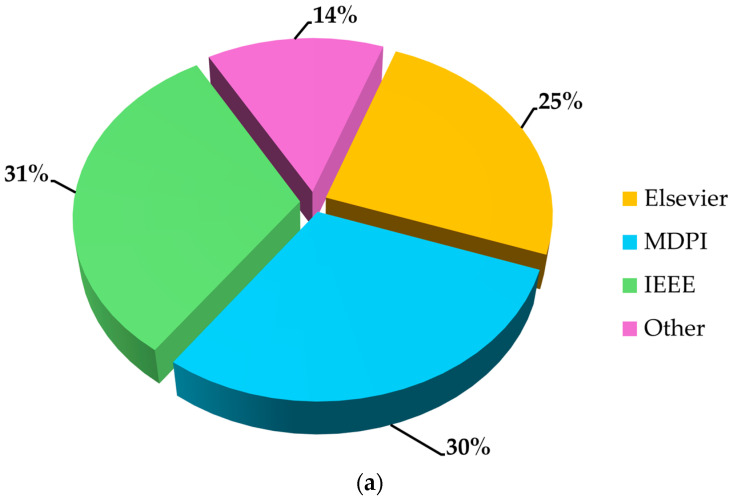

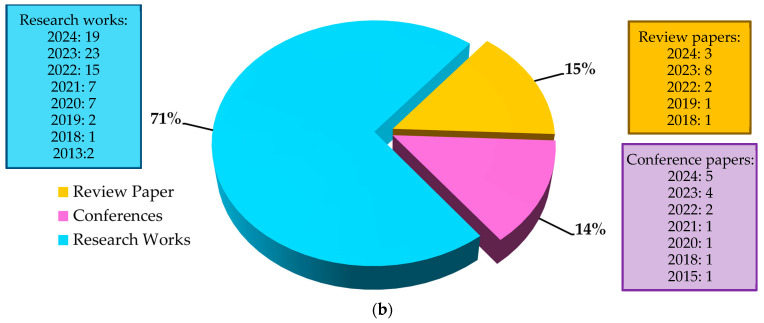
(**a**) Selected articles sorted by publishers, (**b**) selected articles sorted by typology.

To provide an accurate analysis of the topics covered in this manuscript (i.e., on-board systems for driver and vehicle monitoring, and off-board systems for traffic and road management and monitoring), the authors have reviewed 119 documents, including research articles, conference papers, review articles, and websites. The primary sources for these articles were Elsevier, MDPI, and IEEE. In Figure 3a, the selected articles are categorized by publisher, while in Figure 3b, they are sorted by type.

## 2. On-Board Driver Monitoring Systems for Road Safety Applications

**Figure 4:**
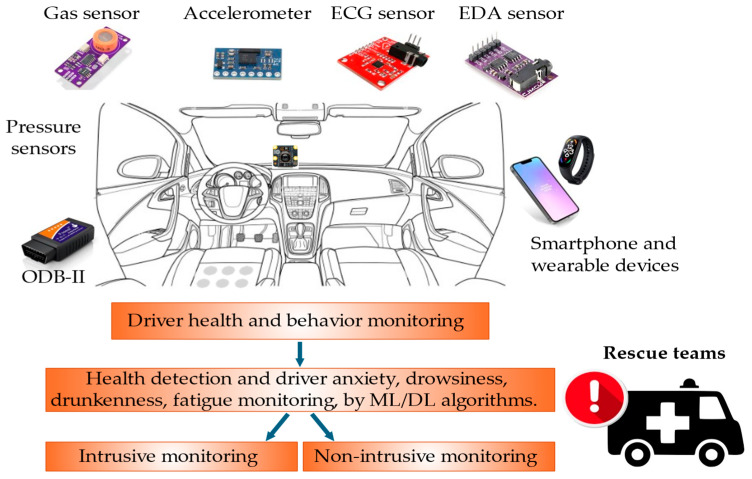
Example of an innovative DMS based on intrusive and non-intrusive methods for the acquisition and processing of biophysical and behavioral driver parameters by using sensors and cameras integrated into the cockpit and wearable devices.

In recent years, road safety has become increasingly important, driven by the need to reduce the number of accidents. In this context, on-board DMSs are emerging as a promising solution to improve the safety of vehicles and occupants [31]. Different methods have been proposed in the literature to detect the driver’s condition, starting from the acquisition of biophysical signals or video/images, allowing the detection of drowsiness, stress, tiredness, and distraction of the driver, i.e., all aspects that reduce the attention to driving and considerably increase the risk of accidents [32]. The primary goal of DMSs is to identify potentially hazardous driving behaviors and provide immediate alerts to the driver; they can also record and analyze the driving data to prevent accidents, promoting a culture of responsible road safety. Figure 4 illustrates key devices used in DMSs, including wearable and non-wearable sensors for collecting biophysical data, and cameras to capture facial features, head movements, and eye behavior, such as blink rates and eyelid drooping. ML algorithms process these data to determine stress, drowsiness, alcohol effects, and the driver’s health condition. A further feature of DMSs is the capability to report hazardous conditions to the driver, authorities, and emergency services. Below, the methodologies proposed in the literature are analyzed: the intrusive methods involve wearable devices, while non-intrusive ones rely on image processing and biosignal acquisition by sensors integrated into the seatbelt, steering wheel, and seat.

Monitoring the driver’s condition is crucial for road safety, enabling early detection of driver anomalies, like drowsiness or drunkenness. These systems, integrated into modern vehicles, not only improve personal safety but also complement the vehicle’s advanced diagnostic systems, which detect malfunctions and analyze driving style to prevent risky situations. In addition, systems for monitoring vehicle stability play an essential role in ensuring road safety, guaranteeing constant control over the vehicle and driving conditions. These technologies, working in synergy, also contribute to a more efficient traffic management, optimizing urban mobility and reducing the risk of accidents thanks to the collection and analysis of data on driving behavior and vehicle conditions.

This section explores innovative DMSs highlighted in recent literature. It is organized into four subsections: driver health monitoring, driver drowsiness monitoring, driver drunkenness monitoring, and comparative analysis of the reported articles.

### 2.1. Driver Health Monitoring Systems to Detect Stress, Anxiety, and Other Biophysical Parameters

This subsection analyzes the applications developed by the authors for monitoring driver health during driving operations. In this regard, Kaur et al. proposed an IoV-Health system dedicated to monitoring the health conditions of the driver [11]. The monitoring occurs with six wearable sensors, such as an Electrocardiogram (ECG) and Blood Pressure (BP), Body Temperature (BT), heart rate (HR), Respiration Rate (RR), and alcohol sensors. Based on the data acquired by the sensors, the system can manage four emergency states: heart failure, stress, drowsiness, and drunkenness. Additionally, the system designed by the authors issues alerts to nearby vehicles, hospitals, and ambulances according to the detected alarm level, ensuring prompt emergency assistance for the driver. The authors used different ML algorithms for the classification of the recorded data, obtaining high percentages of accuracy through Random Forest (RF) (99.86%), ensemble classifier (EC) (99.83%), and decision tree (DT) (99.7%) algorithms. Finally, the authors observed that their system’s efficiency surpassed similar systems by more than 50%.

Affanni et al. presented a smart necklace for monitoring the driver’s well-being [12], including a sensor that monitors HR and blood oxygen saturation (SpO_2_). The collected data are sent to a mobile application via Wi-Fi for continuous real-time viewing. The authors focused on the necklace’s comfort, obtaining excellent results from the driving tests they carried out. The algorithm for the data processing is based on the “Mountaineer algorithm” for pick detection in ECG signals [33]. Gong et al. in [13] adopted remote photoplethysmography (rPPG) based on a monochrome camera to measure HR. They proposed an innovative approach called “Quality-guided Spectrum Peak Screening” (QSPS) to eliminate noise interference related to rapidly changing lighting conditions, head movements, or vibrations of the vehicle. The QSPS framework detects PPG signals on multiple regions of the face, in order to attenuate the residual noise through a spectral peak screening algorithm. With this innovative approach, the authors demonstrated that QSPS has greater robustness than infrared methods, achieving a Mean Absolute Error (MAE) of 4.32 bpm, a Root Mean Square Error (RMSE) of 6.15 bpm, and a Pearson correlation coefficient of 0.943 in the nighttime test and 0.906 in the daytime test for 10 min time monitoring.

Jiao et al. conducted an experimental study for real-time detection of driver fatigue status to improve driving and traffic safety [34]. The authors conducted an experimental study to gather data, including fatigue levels measured using the Karolinska Sleepiness Scale [35], as well as Heart Rate Variability (HRV) and Electrodermal Activity (EDA) parameters. Broad statistical analysis revealed significant variations in several HRV and EDA features across different fatigue levels. Using various ML techniques, the Light Gradient Boosting Machine (LGBM) classifier demonstrated the best performance for binary classification, achieving an accuracy of 88.7% with HRV and EDA features as inputs. For three-class classification, accuracy slightly dropped to 85.6% with the RF classifier. These findings highlight the effectiveness of combining HRV and EDA features to capture diverse physiological responses to fatigue, enhancing overall detection accuracy.

Leicht et al. in [36] evaluated non-intrusive methods for monitoring HR and RR, including a hybrid imaging approach, under both simulated and real driving conditions. The feasibility of these methods was assessed by comparing data from non-intrusive sensors with reference sensors. In laboratory settings, magnetic induction and photoplethysmography sensors, integrated into seatbelts, along with hybrid imaging combining visual and thermal data, were tested for RR detection. In real driving conditions, HR and RR were monitored using hybrid imaging and a seat-integrated capacitive ECG across both urban and rural scenarios. The laboratory tests demonstrated reliable RR detection with all three sensor technologies. In real-world rural driving, both HR and RR were reliably detected. However, only RR detection is feasible in urban driving due to motion artifacts affecting the capacitive ECG, which impairs HR detection.

**Figure 5:**
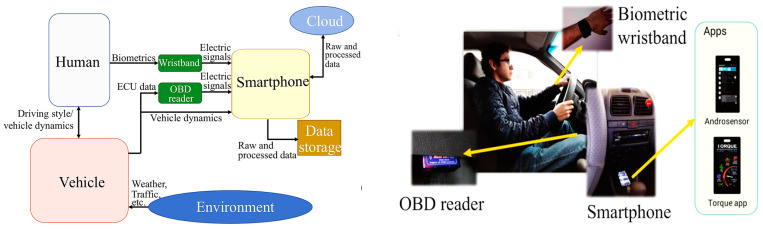
The system proposed in [37] integrates on-board and remote vehicle sensors to develop algorithms that estimate pollutant emissions, fuel consumption, driving behavior, and driver health.

The system proposed in [37] allows driver and vehicle monitoring, stating that the driver affects the vehicle’s performance and vice versa. The proposed system integrates on-board and remote vehicle sensors to develop algorithms that estimate pollutant emissions, fuel consumption, driving behavior, and driver health. The main contribution lies in analyzing the interaction between these factors and between driver behavior and vehicle performance, conducting an experimental analysis with different drivers and routes, and the results are implemented in a mobile application. Unlike commercial systems, this approach utilizes standard sensors and established algorithms tested interactively. During the system design, principal component analysis was employed to reduce the number of variables for training and testing, minimizing Bluetooth data transfer between the biometric wristband, smartphone, and vehicle’s central computer, as shown in Figure 5. Experimental results indicate the system accurately predicts fuel consumption 84% of the time, pollutant emissions 89%, and driving behavior 89%. Notably, the study revealed significant correlations between the driver’s heart condition and traffic conditions.

The model proposed by Škorić in [38] explores the classification of stress levels using capacitive Electrocardiogram (cECG) signals recorded during driving with non-intrusive acquisition systems featuring various hardware configurations. The ML model developed for this purpose relies on four features, all derived from detecting the R peak (the local maximum of the ECG signal), which is recognized as the most reliably identified point even in lower-quality cECG recordings. These features were chosen for their low computational complexity, facilitating real-time application. The model was evaluated using three datasets of driving-related recordings: high-quality ECG captured with direct skin contact electrodes, medium-quality cECG obtained via a portable cushion with electrodes operating through clothing, and lower-quality cECG from car seat-embedded electrodes also functioning through clothing. The model demonstrated very high accuracy: 100% for high-quality ECG, 96.67% for medium-quality cECG, and 98.08% for lower-quality cECG.

### 2.2. On-Board Systems for Driver Drowsiness Monitoring

To mitigate the risk of accidents caused by driver drowsiness, Ebrahimian et al. introduced a novel methodology for multi-level drowsiness detection using Convolutional Neural Networks (CNN) and Long Short-Term Memory (LSTM) networks [39]. This approach relies on HR and Respiratory Rate Variations (RRV) data. The authors tested their model on data from 30 participants in a driving simulation, achieving effective results with the combined CNN and LSTM models. Specifically, they reported an accuracy of 91% for a three-level drowsiness classification and 67% for a five-level classification.

Similarly, Alguindigue et al. conducted research aimed at reducing drowsy-driving-related accidents by monitoring sleep-induced attention loss [40]. To assess driver sleepiness, they explored various indicators, including HRV, percentage of eyelid closure over time (PERCLOS), blink rate and percentage, and EDA signal. They employed three deep learning (DL) algorithms: a Sequential Neural Network (SNN) for HRV, a 1D-CNN for EDA, and a Recurrent Convolutional Neural Network (CRNN) for eye-tracking data. The models using HRV and EDA provided high accuracy, achieving 98.28% and 96.32%, respectively, promoting real-time neuro-adaptive systems to detect driver drowsiness. In [41], a different approach is used to detect driver drowsiness based on the Johns Drowsiness Scale (JDS). This method utilizes Optalert glasses equipped with an IR transmitter/receiver to measure eyelid velocity and blink duration through reflectance oculography. The JDS provides ratings on a scale from 1 to 10, derived by the mean and standard deviation of eyelid movement duration and relative velocity during blinks. According to this, drivers are classified as alert if their score falls between 0 and 4 and drowsy between 5 and 10.

Sukumar et al. proposed a model to determine driver stress and drowsiness by detecting ECG signals, head movements by accelerometers, and hand contact with the steering wheel by interdigitated capacitive sensors [14]. By integrating these data, the authors gained insights into driving behavior; to assess stress level, they analyzed ECG signals using the Synchronized Continuous Wavelet Transform (CWT-SST) to evaluate HRV. The same algorithm was applied to detect drowsiness level through head movement data. Their model provided an accuracy of 91.82% in stress detection and 97.30% in identifying drowsiness level. Amidei et al. developed a model to detect driver drowsiness that provides timely warnings [42]. Their approach uses the Empativa E4 wristband that monitors the skin conductance; moreover, the authors tested three ML algorithms for classifying drowsiness, finding that the RF one performed best, achieving an accuracy of 84.1%.

**Figure 6:**
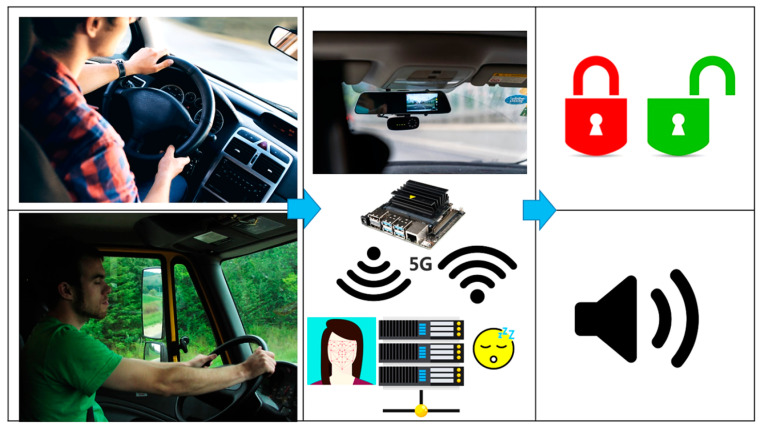
Innovative model for facial recognition and driver drowsiness detection proposed in [43].

To enhance vehicle and driver safety, Díaz-Santos et al. proposed a model combining facial recognition and drowsiness detection [43], which operates in two phases: the first identifies the driver before the vehicle starts, and the second monitors eye activity for fatigue and drowsiness signs (Figure 6). For driver identification, the authors utilized OpenCV, a computer vision library that detects facial features such as eyes, nose, and mouth. Drowsiness detection relies on prolonged eye closure by a CNN model. The system was developed in Python, employing the Keras deep learning framework and OpenCV. The proposed model achieved 100% recognition accuracy under ideal conditions, which decreased slightly to 97.5% when the driver wore glasses. Khan et al. implemented a non-intrusive DMS using computer vision techniques [44]. The proposed framework operates on four levels: an embedded system, edge computing, cloud computing, and a user interface. In the edge computing layer, facial features such as eye, mouth, and jaw movements are analyzed to detect drowsiness signs. The system then sends notifications to relevant authorities, including general information and drowsiness score, to assess the driver’s condition; after testing, the model provided high robustness with 96% overall accuracy.

**Figure 7:**
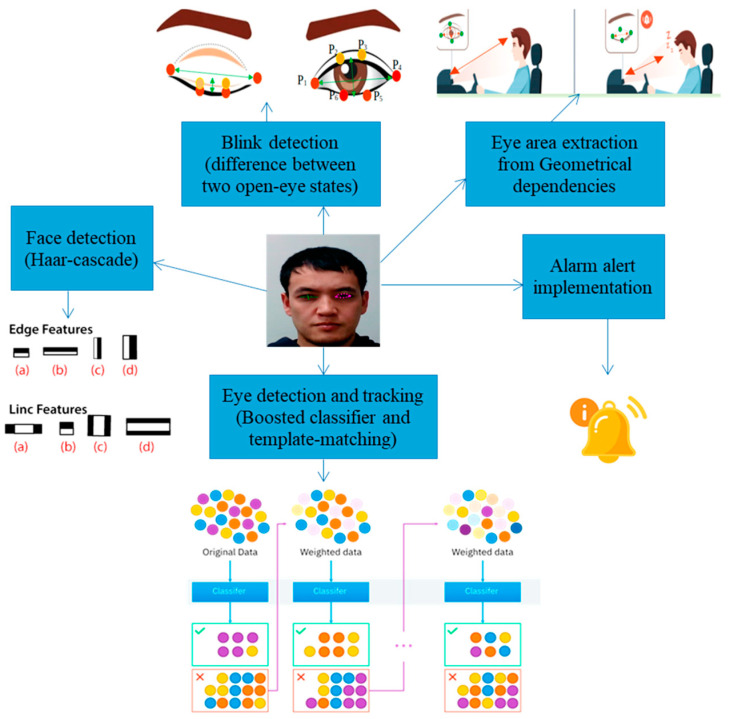
Main features of the method proposed by the authors in [15]; the Haar–Cascade classifier was trained to detect faces and extract the related features (a–d). After detection, the authors captured the coordinates of facial landmarks and exported them to a comma-separated value (csv) file, based on the proposed classes.

Safarov et al. in [15] applied a combination of deep learning (DL) and computer vision algorithms to detect driver drowsiness. They trained their model using custom datasets and tested it with multiple subjects. Eye-blink frequency and mouth region coordinates were captured using facial landmarks (Figure 7). These landmarks were analyzed in real time to monitor fluctuations in eye-blinking rates and mouth movements. Their real-time experiments revealed a correlation between yawning and prolonged eye closure, which were classified as signs of drowsiness. The model demonstrated strong performance, achieving 95.8% accuracy for detecting closed eyes, 97% for open eyes, 84% for yawning, 98% for detecting right-sided head falling, and 100% for left-sided head falling. Additionally, the system enabled real-time eye state analysis, effectively classifying eyes into “Open” or “Closed” categories based on threshold values.

**Figure 8:**
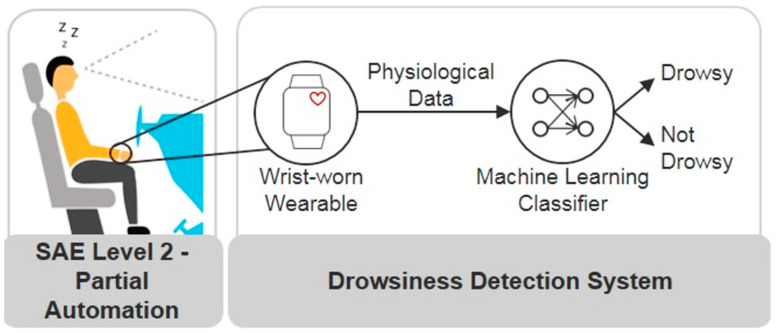
Architecture proposed in [45] for non-intrusive monitoring of the driver to detect the state of drowsiness by processing the ECG signals via ML algorithms.

The proposed approach in [15] may not be suitable for drivers wearing sunglasses. Sunglasses can obstruct the model’s ability to accurately detect eye landmarks, which are crucial for measuring eye blinks and assessing driver alertness. Building on the concept of non-intrusive monitoring, Kundinger et al. developed an ML approach for detecting driver drowsiness using physiological data from a sensor embedded in a wrist-worn bracelet (Figure 8) [45]. They compared the bracelet’s performance against a medical-grade ECG device. Among the binary classification algorithms tested, the K-Nearest Neighbor (KNN) model achieved the highest accuracy at 92.13%.

Vyas et al. introduced the “DriverSense” system, designed to enhance the safety of drivers on two- or three-wheeled vehicles [46]. The framework relies on a wearable device equipped with mobility sensors (gyroscope, accelerometer, GPS) and biosensors able to collect real-time biosignals (EEG-electroencephalogram, PPG, ECG, SpO_2_). By combining the detected signals, the designed system can assess the driver’s stress level and driving behavior in real time, classifying the first as normal or stressed and the second one into three categories: slow, normal, and aggressive. The authors reported classification accuracies of 86.05% for driving behavior and 88.24% for stress detection.

### 2.3. On-Board System for Driver Drunkenness Monitoring

**Figure 9:**
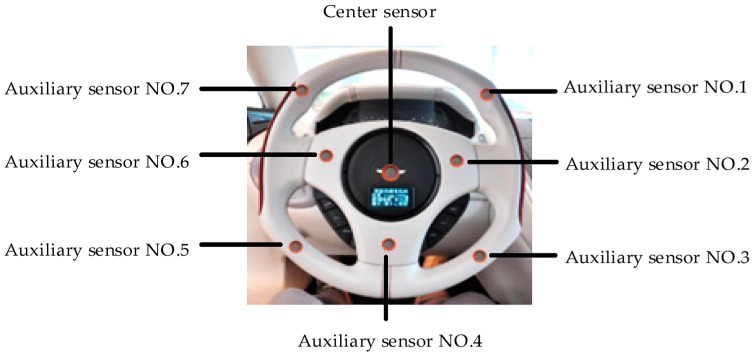
MQ3 gas sensor array integrated into the steering wheel used for the model proposed by the authors in [48]. They also integrated an Organic Light-Emitting Diode (OLED) display screen showing the alcohol concentration level, indicating the level of drunkenness.

Drunkenness is a significant cause of traffic accidents. To address this issue, Ku-mar et al. proposed an IoT-based system designed to detect driver intoxication and prevent the car engine from starting [47]. Their model utilizes advanced sensors to detect alcohol on the driver’s breath (using an MQ-3 sensor), a Raspberry Pi microcontroller, and a Global Positioning System (GPS) module for tracking the vehicle’s location. When the driver attempts to start the car, the system checks for alcohol levels. If the detected level exceeds legal limits, the microcontroller activates a buzzer, warning the driver that the engine will shut off shortly. Simultaneously, a Global System Mobile (GSM) module sends the vehicle’s position to a monitoring center or designated contacts. Similarly, Liu et al. developed an intoxication detection system integrated into the vehicle’s steering wheel for improved data accuracy [48]. This system uses an array of sensors, as shown in Figure 9, and processes the data through a weighted fusion algorithm. If the sensors detect signs of intoxication, a signal is sent to the microcontroller, which then acts on the ignition relay to prevent the engine from starting.

Swarna et al. proposed an architecture known as the Improved IoT-based Driver Safety Model (IIoTDSM) to prevent driving under the influence of alcohol [49]. This system integrates alcohol detection sensors into the vehicle’s steering wheel. Key components include a Wi-Fi module, an Arduino microcontroller, an MQ3 gas sensor, and a blink sensor. If the system detects that the driver’s Blood Alcohol Concentration (BAC) exceeds legal limits, it prevents the vehicle from starting and alerts the appropriate authorities.

**Figure 10:**
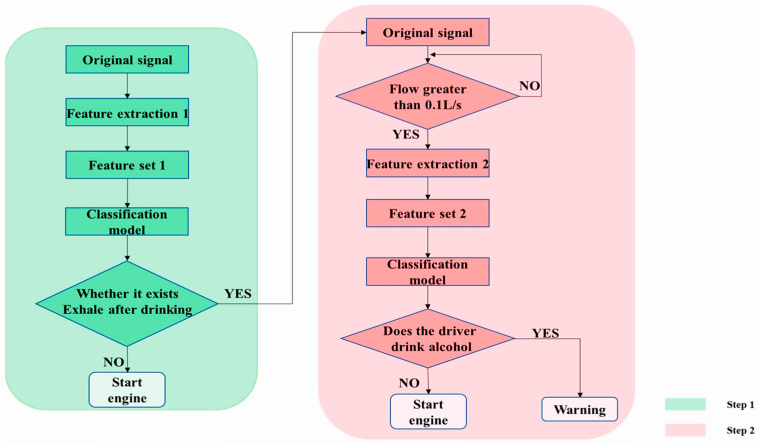
Flowchart of the model proposed in [50], which checks whether the driver has drunk alcohol, preventing the engine from being started if the outcome is positive.

In response to the rise of shared car usage, Wang et al. developed a model to detect intoxicated individuals inside the vehicle or determine if the driver is drunk [50]. Their system features an electronic nose equipped with gas sensors to measure alcohol concentration in the vehicle’s air. The detection process involves two steps: first, assessing the overall alcohol level in the car, and second, determining if the driver is intoxicated. These steps are illustrated in Figure 10. The authors reported high accuracy, achieving 99.44% in the first and 100% in the second steps, with a sampling time of just five seconds.

In [51], the MQ-3 sensor is used to measure BAC through the driver’s breath and surrounding air, comparing the readings with the legal limits in respective countries. The system includes a proximity sensor to prevent the MQ-3 sensor from being covered. If drunkenness is detected, a flashing red light alerts the driver and the vehicle will not start. Furthermore, Cho et al. conducted a study using chromatography to detect BAC and assess the drunkenness level [52]. Although this method is more complex, it offers an alternative to semiconductor sensors, overcoming the short duration limitations and lack of stability.

### 2.4. Comparative Analysis of On-Board Driver Monitoring Systems

The literature analysis has shown that the primary challenge lies in accurately detecting anomalies in driver health, drowsiness, and intoxication using intrusive and non-intrusive methods. Various approaches are discussed to assess the driver’s condition, involving monitoring biophysical parameters such as heart rate (HR), Heart Rate Variability (HRV), Respiratory Rate (RR), and Electrodermal Activity (EDA), and facial expressions such as blinking frequency, eyelid closure duration, and excessive head movements.

The methods examined in Section 2.3 focus on measuring BAC from breath analysis by MQ-3 sensors, preventing the engine from starting if the limit is exceeded. Advanced systems also employ cameras to analyze head movements or facial expressions to enhance accuracy. Various ML algorithms, such as Random Forest (RF) and Convolutional Neural Networks (CNN), are commonly used to assess the model accuracy, with CNNs particularly suited for real-time image analysis. The reviewed methodologies demonstrated high accuracy, indicating their readiness for commercial implementation. A crucial feature of these systems is maintaining driver comfort, ensuring that monitoring does not interfere during driving. Non-intrusive approaches using cameras or sensors integrated into belts or seats are preferred, as they preserve comfort despite requiring higher computational power. Ongoing research aims to enhance the DMS accuracy in assessing health conditions, for instance by combining biophysical data, postural changes, and driving behavior. In this regard, inertial sensors on the steering wheel can measure the steering force, piezo-resistive sensors in the seatbelt can monitor the respiratory activity, and the driver’s posture can be monitored by pressure sensors in the seat and the stress level by ECG electrodes placed on the steering wheel.

Section 5.1 discusses commercial DMSs that detect drowsiness and distractions, which are major contributors to accidents. Table 1, Table 2 and Table 3 summarize the key features of the reviewed studies, including applications, data acquisition devices, monitored parameters, intrusiveness levels, alarm systems, ML algorithms used, and accuracy rates.

Future challenges in the field of driver health monitoring concern various technological, ethical, and practical aspects to achieve the following goals:

- High accuracy and reliability of parameter detection by using non-intrusive devices integrated into the vehicle and wearable devices;
- Integration with embedded ML/DL models to process large data amounts in real time;
- The development of systems that work in synergy with vehicle diagnostics, autonomous driving, and traffic management solutions;
- Privacy and high security of user data against cyber-attacks and unauthorized use.

Technological advancements are pushing toward systems implementing multi-modal analysis for detecting and simultaneously processing driver, vehicle, and traffic data. Recently several sensing technologies have been proposed in the literature for discretely detecting the driver’s biophysical parameters, addressing critical aspects of safety and well-being in the driving context. Flexible piezoelectric sensors offer a promising approach for real-time posture analysis; their integration into car seats enables continuous monitoring without being intrusive to detect fatigue or discomfort early on [53]. Also, radiofrequency and optical signal reflection can be exploited to extract driver biophysical parameters; radar and LiDAR devices have demonstrated capability in measuring respiratory and cardiac parameters (e.g., HR, HRV), providing accurate and real-time physiological data while maintaining driver comfort [54]. Finally, cameras installed in the vehicle cockpit can be used to measure heart and respiratory parameters; for example, estimating HR using images acquired from an iPPG (imaging PPG) camera is a novel and non-invasive technique, particularly useful in dynamic environments like vehicles, as it avoids direct contact with the driver [13]. These approaches highlight the potential of combining different sensing modalities to create a robust and comprehensive DMS. Future work could focus on integrating these technologies into a unified platform, addressing challenges such as data fusion, reliability in varying conditions, and ensuring user privacy.

## 3. Smart Monitoring Systems Based on Vehicle Information

**Figure 11:**
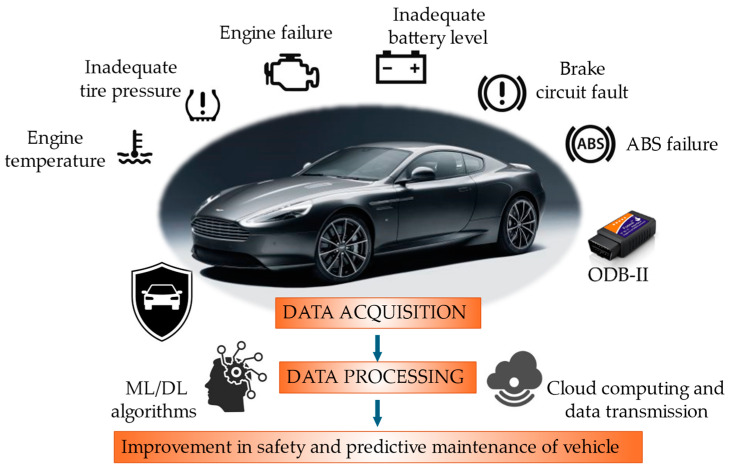
Vehicle monitoring based on ML algorithms for processing data acquired by sensors for detecting and predicting vehicle anomalies.

In recent years, vehicle safety has become a priority in the automotive industry, leading vehicle manufacturers to develop and implement advanced technologies for monitoring and enhancing road safety. These systems protect vehicle occupants while improving interaction with the surrounding environment. One key safety feature is the Tire Pressure Monitoring System (TPMS) [55], which alerts drivers to under- or over-inflated tires, improving vehicle safety. Additionally, sophisticated systems can assess road conditions in real time, detecting hazards such as potholes, ice, or other surface irregularities. Driver-assistance technologies, including stability and braking control, employ sensors and cameras to continuously monitor vehicle performance and the surrounding environment, and automatically intervene in critical situations, providing a more controlled and safer driving experience. These advancements are often integrated into broader smart mobility strategies, where connectivity and data collection are crucial for improving road safety and minimizing accident risks. Such innovations contribute to a safer, more informed, and more responsible driving experience. A comprehensive vehicle monitoring model relies on engine and battery health data, tire pressure and wear, and oil and coolant temperatures, all collected via the on-board diagnostics (OBD-II) system and then processed by ML algorithms to assess the vehicle safety level, as illustrated in Figure 11. Furthermore, this information allows predictive maintenance to guarantee optimal vehicle performance and efficiency.

Integrating vehicle status information with driver and road conditions can significantly improve driving safety by creating a holistic and reactive system that can prevent accidents and optimize risk management. Through vehicle diagnostic tools and driver monitoring, it is possible to achieve adaptation to a driving style; furthermore, by detecting fatigue or drowsiness signs, the diagnostic system can reduce the vehicle’s speed until it stops. Also, automatic adaptation of vehicle parameters can be performed based on real-time information on road conditions (e.g., slippery pavement, heavy traffic, or sharp curves) by means of the driving assistance systems, such as traction control and stability, and by warning the driver to slow down in case of dangerous road conditions or brake/suspension failures.

### 3.1. On-Board Smart Monitoring Systems for Vehicle Diagnosis

The first approach for monitoring vehicle conditions uses intelligent diagnostic systems that detect anomalies and malfunctions before the failure occurs. Yang proposes a model to enhance fault diagnosis in electric vehicles by a system based on the Controller Area Network (CAN) bus and diagnostic communication architecture compliant with the AUTOSAR standard [18]. This approach aims to improve software reusability and the portability of fault diagnosis systems for electric vehicles. By leveraging AUTOSAR, developers can focus more on functionality rather than system architecture. The system provides a fault detection response time of 0.0217 s and a detection accuracy of 98.70%.

Kim et al. developed a diagnostic algorithm to effectively identify and localize faults of a 4WIS4WID vehicle which, employing redundant hardware and fault-tolerant controls, allows continuous operation even in the presence of faults [56]. The limited degrees of freedom compared to the number of potential failures complicate the data analysis process. The algorithm uses residual analysis to compare vehicle behavior with sensor data; these residuals are converted into three-channel images using histogram analysis and logical functions to train a CNN algorithm to detect and locate faults. The Recursive Least Squares (RLS) method also classifies fault types and vehicle output in response to detected issues.

Kannan et al. proposed a fault diagnosis system using a PIC microcontroller within a Predictive Maintenance (PdM) framework, highlighting the crucial role of extending vehicle life [57]. The system collects and processes engine, transmission, and control unit data, utilizing IoT and ML technologies to monitor vehicle health and accurately identify faults. The system performs better than existing approaches by generating detailed fault information and enabling more effective and proactive vehicle management.

Min et al. developed a framework focused on sensors’ self-diagnosis, as a sensor is reliable when it works correctly [58]. Their approach uses a residual consistency-checking algorithm to identify and isolate faulty sensors by leveraging their redundancy. The system employs a Denoising Sparse Autoencoder (DSAE) integrated with a soft threshold-shrinking block, enhancing feature representation and anomaly detection accuracy. Experimental results confirm that the developed solution effectively detects and isolates sensor faults, contributing to more robust and reliable monitoring.

**Figure 12:**
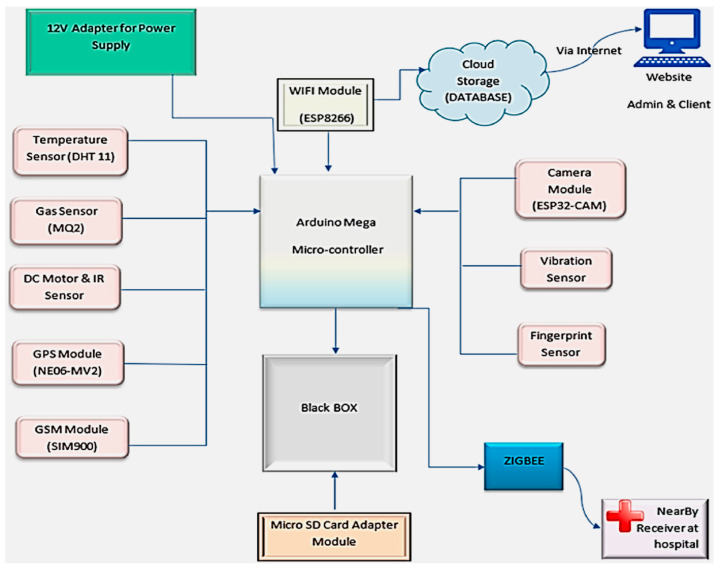
Architecture of the vehicle monitoring system proposed in [59].

To enhance accident prevention, Shermila et al. proposed an automotive black box that continuously monitors and records data from on-board sensors to uncover the actual causes of accidents [59]. This system provides information, such as speed and acceleration during and after an accident, to verify if safety devices functioned correctly in case of impact; it integrates different sensing devices, including collision sensors to detect the accident, temperature and gas sensors to identify anomalies derived from the impact, and GPS to determine the vehicle’s position. In case of emergency, sensor data are transmitted to law enforcement and medical services via GSM and an internet network. Figure 12 illustrates the system architecture through a block diagram.

### 3.2. Innovative Systems for Detecting Vehicle Instability Behavior While Driving in Adverse Asphalt Conditions (Icy Asphalt, Uneven Asphalt, and Aquaplaning)

Driving in difficult environmental conditions poses significant risks, especially at high speeds. Ensuring vehicle stability on icy or wet roads, where the lack of friction between asphalt and tires leads to the loss of the vehicle’s control, is essential to increase driver safety. Fichtinger et al. [60] developed a method combining various aquaplaning detection models, including monitoring of the slip slope variations (similar to Gustafsson’s work [61]), changes in rolling resistance, wheel spin-up/down detection, and analysis of wheel speed variance, acceleration, and rolling resistance using CUSUM (Cumulative Sum Control Chart). Their approach utilizes a minimal set of sensors, focusing on Electronic Stability Control (ESC) data and driving torque.

Hu et al. propose an advanced system for identifying slippery road conditions using Connected Vehicle (CV) technology [22]. Their model leverages data from connected vehicles and employs deep learning algorithms, particularly LSTM networks. These networks, trained with simulated data from VISSIM software and optimized using Bayesian algorithms, accurately classify road conditions into dry, snowy, and icy categories, achieving Success Rates (SRs) of 100%, 99.06%, and 98.02%, respectively. A key innovation is the system’s ability to improve driving behavior: issuing timely warnings helps drivers adjust speed and maintain safe distances, reducing potential accidents by up to 90%. The system effectively detects black ice, a critical factor in preventing hazardous driving incidents.

Lee et al. [62] developed an advanced black ice detection system for autonomous vehicles, notoriously difficult to detect, by using CNN algorithm to identify it accurately. To enhance the system’s performance, the authors applied data augmentation techniques, training the CNN with images representing various road conditions prone to black ice formation. The model achieved a detection accuracy of 96%, showing high efficacy to reduce accidents in icy conditions. Another critical issue affecting urban roads is the presence of potholes and cracks, which can make driving uncomfortable and damage vehicles. Bhadraray et al. addressed this problem by creating a model that adjusts vehicle speed and trajectory to avoid potholes while maintaining the travel route [63]. They simulated the system using a TurtleBot3 Burger robot equipped with an RGB camera and trained the YOLOv4-Tiny model on a dataset of 2160 images to detect potholes. Additionally, they used Canny edge detection to identify road boundaries within the region of interest.

Raja et al. introduced the Smart Pothole Avoidance Strategy (SPAS) that uses the Deep Deterministic Policy Gradient (DDPG) algorithm for dynamic pothole management [64]. A key innovation of SPAS is its hybrid recognition model, combining audio and visual feedback from passengers through a mechanism called Speech and Gesture (HRM-SG). This interactive feedback refines the DDPG system’s capabilities, enhancing pothole avoidance accuracy and improving passenger comfort. SPAS optimizes lane changing and speed adjustments based on sensor data and passenger input, making travel safer and more comfortable. Experimental results show significant improvements over existing methods with a 10–15% increase in pothole avoidance accuracy, a 10–12% boost in comfort, and 8–10% faster convergence. This work highlights advancements in autonomous vehicle technology and the value of integrating human feedback to optimize navigation performance.

### 3.3. Smart Systems for Driver Style and Behavior Monitoring

**Figure 13:**
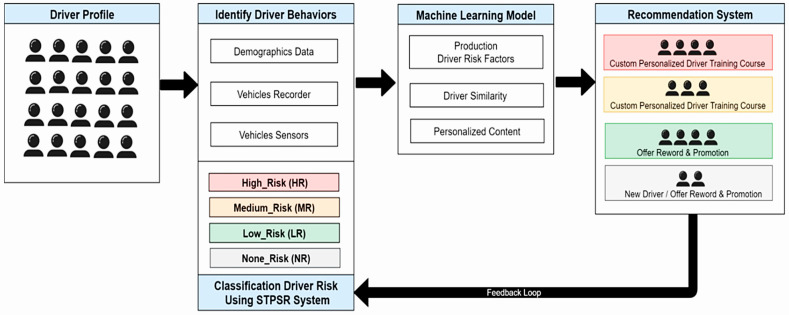
Architecture proposed in [66] based on different functional blocks.

Sensor data acquisition enables the identification of a driver’s behavior, distinguishing aggressive and irrational styles [65]. Nouh et al. introduced *SafeDriver*, a Dynamic Driver Profile (DDP) system, which uses historical data on traffic violations and accidents to classify drivers into four risk levels [66]. The goal was to improve driver behavior and road safety by alerting other vehicles connected through the IoV. The authors derived driver profiles using data from the Saudi Traffic Points System Regulation (STPSR) and applied DL techniques to process them. Figure 13 shows the architecture for driver classification.

Similarly, Mohammed et al. developed a real-time driving style recognition model using electronic monitoring devices to detect the vehicle speed, engine RPM, coolant temperature, and geolocation coordinates [9]. These data are transmitted to a server, visualized through a virtual dashboard, and stored as driving history and route records. Shichkina et al. proposed an algorithm that processes twenty descriptive features to monitor driving behavior [67]. By accessing data from the diagnostic system (OBD-II), the model gathers information on environmental conditions, driver actions, and vehicle performance, to be analyzed by a Kohonen network, which clusters drivers based on driving style and associated risk levels. Chhabra et al. developed a federated learning approach for monitoring driver behavior, where data from connected vehicles enhance the algorithm performance [68]. By using CNN-LSTM and CNN-Bi-LSTM deep learning models, they analyzed data collected from aboard-smartphone sensors and in-car devices to classify driver behavior accurately. In another study, Malik et al. focused on analyzing driving patterns using real-world data gathered via the vehicle OBD port [69], employing hierarchical clustering, k-means, ANN, and multilayer perceptrons to identify driving behavior patterns and categorize data into meaningful groups. A key innovation is the use of Inter-Class-ReLU activation function, which strengthened the model’s robustness and classification accuracy.

Lattanzi et al. explored driver behavior detection using data from on-board sensors [70]. By applying Support Vector Machines (SVM) and a feed-forward Neural Network (NN), they extracted descriptive features to identify driving patterns, enhance road safety, and prevent accidents caused by risky behaviors. Dong et al. detected fatigue and distracted driving by vision-based techniques and ML models [71]. For fatigue detection, they used facial feature analysis by the RF algorithm to assess driving conditions; instead, to classify distracted driving behaviors, the authors implemented a CNN model, obtaining 91% and 97.5% accuracy values, respectively.

### 3.4. On-Board Innovative System for Tire Monitoring

Vasantharaj et al. proposed an innovative indirect TPMS that leverages existing vehicle sensors to measure tire pressure indirectly [55]. Their model also detects grip conditions and pressure loss based on speed, enhancing overall performance monitoring.

Màrton et al. introduced an advanced indirect TPMS (iTPMS) that uses modern signal processing techniques combined with a CNN for detecting pressure-related eigenfrequencies [21]. By integrating wavelet-Fourier transforms with CNN-based pattern recognition, their approach demonstrates superior accuracy and efficiency in experiments, marking a significant step forward in AI-driven TPMS technologies.

Shan et al. presented another indirect TPMS utilizing wheel speed sensors and on-board hardware [72]. They detail a method that processes wheel speed signals through denoising, rim error filtering, and resampling. By analyzing wheel speed spectra and tire vibration characteristics, the system accurately identifies pressure-related frequencies.

Huo et al. developed a tire pressure warning system using magnetoelectric wheel speed sensors to collect and analyze tire speed data [73]. This system, based on beam and frequency techniques, allows early detection of dangerous conditions. Experimental results show improved accuracy and stability compared to traditional methods. Focusing on tire–road interaction, M. Pomoni investigated factors influencing tire–asphalt contact and advocates for the use of intelligent tires [74]. These advanced tires enhance driver perception and responsiveness, improving safety in varying road conditions.

Yu et al. proposed a tire wear monitoring system that goes beyond the traditional methods [75]. Using a magnetic angular velocity sensor operating at 600 Hz and a mini host device for data transfer, they apply the Pacejka model to analyze tire behavior. Data features are extracted by a transformer-based encoder followed by classification through a fully connected layer to define the tires’ wear degree, achieving a 97.77% accuracy, outperforming the Recurrent Neural Networks (RNNs) and SVMs by 5.13% and 4.75%, respectively, even at low sampling rates.

### 3.5. Innovative Emissions Monitoring Systems

Focusing on vehicle emissions control, Anusha et al. introduced a cloud-based model for monitoring and analyzing pollutant emissions using neural networks (NNs) [76]. Their proposed architecture enables real-time emissions tracking and alerts drivers to emergencies. The system gathers data from air quality monitoring stations, vehicle-installed sensors, and remote sensing devices. These data undergo preprocessing, including handling missing values and removing outliers, to create a robust dataset for neural network training. Once processed, the model extracts key features such as vehicle type, engine specifications, maximum speed, and road conditions.

Sao et al. present an innovative method to predict emissions of carbon dioxide (CO_2_), nitrogen oxides (NO_x_), and carbon monoxide (CO) from diesel vehicles using ANN [77]. They evaluate six operational parameters (vehicle speed, engine speed, engine torque, coolant temperature, air/fuel ratio, and intake airflow) collected via on-board diagnostics (OBD) as predictors. The study finds that predictive accuracy improves with additional parameters, though the extent varies depending on which parameters are included. Moreover, the accuracy differs based on vehicle type, with models featuring after-treatment devices showing lower predictive precision compared to those without.

**Figure 14:**
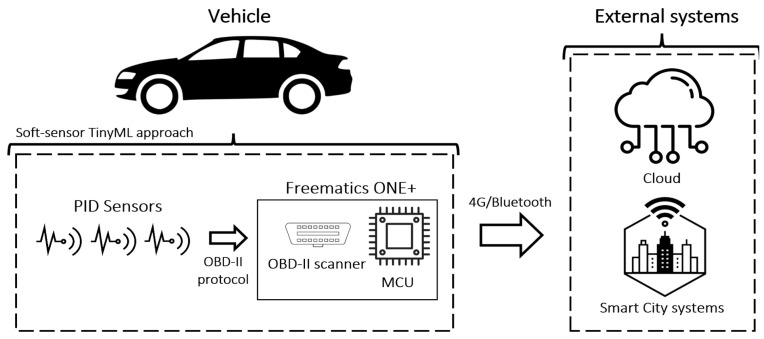
Emissions monitoring architecture proposed in [78].

Andrade et al. propose a continuous emissions monitoring system using the OBD-II interface to indirectly measure vehicle emissions [78]. To enhance data accuracy, they developed a soft-sensor approach that processes engine combustion metrics such as fuel injection and airflow to estimate CO_2_ emissions. Two distinct algorithms, each with tailored mathematical formulations, handle input-specific data. Additionally, an unsupervised TinyML method removes outliers, improving sensor accuracy without relying on cloud connectivity. The results confirm the system’s viability, providing emissions measurements in gCO_2_/km. Figure 14 illustrates the emissions monitoring model developed by authors.

### 3.6. Comparative Analysis of Smart Monitoring Systems Based on Vehicular Information

Monitoring vehicle conditions and driver behavior is essential in reducing road accidents, controlling emissions, and enhancing driver safety. The first step is to establish a diagnostic architecture connected to vehicle one, by integrating redundant hardware, ML algorithms capable of solving multiple simultaneous faults, and self-diagnostic systems for sensors in order to identify faults and enable predictive maintenance. Table 4 highlights the key features of the diagnostic systems analyzed in the literature.

Driving on slippery roads, e.g., covered with ice or water, significantly reduces driving safety, because the reduced grip can lead to losing vehicle control; thus, developing systems that can detect hazardous conditions is essential. The systems discussed in Section 3.2 can identify aquaplaning conditions by detecting wheel slippage or icy roads using cameras and DL algorithms trained on web images and real-time road photos. Additionally, cameras installed on the vehicle can detect potholes on the road, which, when combined with speed modulation and trajectory modification systems, contribute to innovative safety solutions. Table 5 summarizes the key features of the systems analyzed.

Monitoring and analyzing driving styles have become essential for enhancing road safety and optimizing vehicle management. The proposed solutions for monitoring driving behavior and improving safety are promising, incorporating advanced technologies such as deep learning (DL), federated learning, and clustering. However, each approach has limitations that could be addressed through innovations in sensors, data processing, and optimization of predictive models. Future improvements could focus on leveraging mobile technologies for data collection, integrating more advanced sensors for more precise detection of driving styles, and evolving ML algorithms to better accommodate various driver behaviors and environmental factors. Furthermore, the use of a monitoring box, which records the driving data, communicates the vehicle’s position, and alerts emergency services in case of accident, as outlined in [59], is vital. Table 6 summarizes the key features of the driving style monitoring systems analyzed in the literature.

Recent research on tire pressure monitoring systems (TPMSs) has led to innovative approaches that enhance vehicle safety, reliability, and operational efficiency by integrating advanced technologies and intelligent methods. Some proposed models utilize existing vehicle sensors to detect tire grip and pressure conditions, which, combined with ML algorithms such as CNNs, offer a valid solution to reduce the need for additional hardware, also providing accurate results. However, challenges such as computational complexity, sensitivity to variable driving conditions, and reliability in complex scenarios remain. For this purpose, it will be necessary to ensure consistent performance in different operating conditions, reduce hardware requirements for broader accessibility, and enhance integration in new vehicles. This can be achieved by developing more robust, interference-resistant sensors and optimizing AI algorithms in software solutions. Table 7 summarizes the main characteristics of the tire monitoring models discussed in this section.

Recent research on vehicle emissions monitoring and forecasting has shown significant advancements. The solutions proposed by Anusha et al., Sao et al., and Andrade et al. represent crucial steps toward more intelligent and responsive emissions monitoring systems. These approaches stand out for their integration of advanced technologies such as cloud computing, neural networks, and Machine Learning (ML), which enable real-time monitoring and accurate forecasting. However, to enhance reliability, scalability, and accuracy, certain challenges remain, including input data quality, the complexity of forecasting models, and compatibility across various vehicle types. Future improvements could focus on optimizing data models, integrating advanced sensor fusion techniques, and adapting the system to accommodate a broader range of vehicles and operating conditions, ensuring more precise and efficient emissions monitoring. Table 8 summarizes the key features of the emissions monitoring models from the reviewed articles.

Section 5.2 examines commercially available devices for monitoring vehicle conditions, including innovative anti-collision devices and intelligent sensors.

As highlighted in Table 4, Table 5, Table 6, Table 7 and Table 8, most of the systems developed for monitoring vehicle conditions, emissions, tires, and driving style are connected to the vehicle’s OBD-II that provides a lot of parameters such as speed, engine temperature, engine speed, transmission data, and more. When combined with ML methods, these data enhance the effectiveness of monitoring systems. Additionally, OBD-II enables the detection and diagnosis of mechanical issues by storing error codes that can identify specific faults, allowing for timely interventions and reducing the risk of significant damage. A monitoring system that integrates with OBD-II can also help ensure lower emissions and regulatory compliance. In terms of driving safety, OBD-II-based systems can provide valuable insights, such as detecting dangerous driving behaviors or responding to emergencies. In summary, connecting to the OBD-II system offers access to a rich, standardized data source that can greatly enhance the capabilities and effectiveness of vehicle monitoring systems.

Vehicle monitoring systems face several challenges, concerning technological, operational, economic, and regulatory aspects, including the following:

- Providing high accuracy and reliability by sensors used for a long time, comprising the development of self-diagnosis models to verify their correct functioning;
- Ensuring the interoperability and integration with other vehicle systems, given the heterogeneity of technologies and standards between the different manufacturers;
- The management and processing of large amounts of data to develop predictive models to identify possible failures, the control of emissions, or tire wear;
- To guarantee data protection from cyber-attacks;
- The containment of system costs.

In the future, it will be necessary to develop systems capable of performing global monitoring of vehicle conditions, including the prediction of the failure chain, such as suspension wear due to worn tires. Regarding systems to detect driving style, the future is complex, involving the development of software solutions to adapt the vehicle parameters in real time and automatically to driving style by accessing the vehicle diagnostic system (OBD-II), thus limiting the driver’s control.

## 4. Environmental, Road, and Traffic Conditions Monitoring

**Figure 15:**
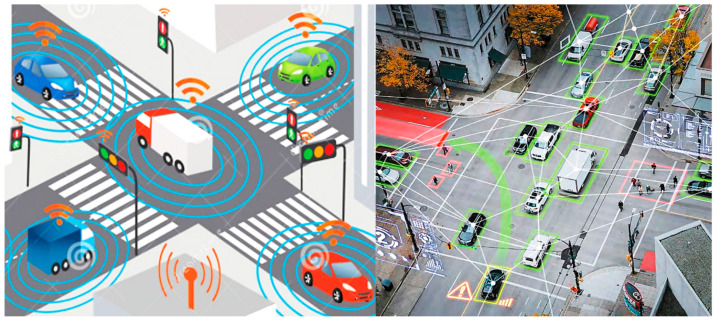
Architecture of a smart city highlighting the interconnectivity between vehicles, infrastructures, and pedestrians within an urban center.

In recent years, Intelligent Transportation Systems (ITSs) have become increasingly significant in the context of urban mobility and traffic management. Central to this innovative approach is the real-time monitoring of traffic flow and environmental conditions. By tracking traffic, authorities can gather vital data on vehicle behavior, congestion, and accidents, enhancing road infrastructure management and ensuring greater safety for road users [79]. Similarly, monitoring environmental factors like air quality and weather conditions is crucial for driver safety and comfort and for promoting sustainable mobility. Together, these aspects support the development of intelligent solutions that streamline traffic, reduce emissions, and elevate the urban quality of life. ITSs, therefore, stand as a key element for future mobility. Figure 15 illustrates how smart cities can enhance traffic management by integrating vehicles, infrastructure, and pedestrians.

Road and traffic conditions have a significant impact on driver and vehicle health, affecting overall safety and performance; complex roads, heavy traffic, delays, roadworks, and poor roads can significantly increase levels of stress and fatigue as well as distraction and nervousness, increasing the risk of accidents. Traffic conditions and poor roads cause faster vehicle wear (e.g., of tires, suspension, and shock absorbers), as well as continuous stop-and-go on congested roads causing thermal stress to the engine and increased brake wear. Difficult road and traffic conditions produce negative effects on the driver (stress, fatigue) and the vehicle (wear, malfunctions), creating high-risk situations for accidents.

### 4.1. Smart Systems for Traffic Monitoring

In the context of road monitoring, Lilhore et al. designed and implemented an Adaptive Traffic Management (ATM) system utilizing IoT technologies and ML algorithms to tackle issues such as congestion, pollution, and delays in urban transportation [25]. This system consists of three core components (vehicles, infrastructure, and events) and applies various scenarios to address diverse transportation challenges. It employs the DBSCAN clustering method to detect anomalies and dynamically adjusts traffic lights based on traffic volume. Experimental results indicate that ATM surpasses traditional traffic management methods by reducing waiting times, alleviating congestion, and enhancing road safety, thereby contributing to smarter transportation planning in urban environments.

Saleem et al. developed an intelligent traffic congestion control system, known as FITCCS-VN, specifically designed for smart city vehicular networks (VNs) [80]. This system leverages ML techniques to analyze and manage increasing traffic congestion and accidents. Collecting data on traffic flow and route availability facilitates more efficient navigation and reduces congestion. The results show that the model achieves 95% accuracy with a 5% error rate, outperforming previous approaches and offering innovative services to help drivers optimize traffic flow. Traditional ML algorithms require the analysis of sensitive user data, posing privacy risks; Federated Learning (FL) provides a solution by ensuring data security during training. In Ref. [81], the authors introduced a cooperative edge caching scheme for next-generation vehicular networks that combines an elastic FL algorithm for personalized content prediction using an adversarial autoencoder (AAE), and a multiagent deep reinforcement learning (MADRL)-based approach for collaborative caching among SBSs (Small-Cells Base Stations), optimizing the cost and efficiency. Experimental results show improved cache hit ratio, personalized predictions, and reduced operational costs compared to traditional schemes.

Mandal et al. proposed an automated real-time traffic surveillance system employing Deep Convolutional Neural Networks (DCNNs) and a graphical user interface [82]. Their system aims to simplify the tasks of human operators in traffic management centers, enabling quicker and more proactive responses to mitigate accidents and congestion. Using an extensive video database, the model detects traffic jams, tracks stationary vehicles, and counts vehicles by applying pixel-level segmentation and object detection techniques. The results demonstrate that the system performs effectively under various environmental conditions, maintaining reliability even with reduced visibility or adverse weather.

For traffic management in Riyadh, Humayun et al. propose an intelligent traffic management system that integrates IoT, cloud computing, 5G, and big data to enhance real-time traffic efficiency [83]. The system gathers data on road conditions and accidents and then communicates alert messages. This model is structured into three levels. In the first level, the Information Acquisition one, data are collected via vehicle-mounted sensors that capture images and roadside infrastructure that monitors vehicle counts. In the second Network level, information is transmitted over a 5G Wi-Fi network to the cloud for processing. In the third level, the Application Level, dashboards integrate Google Maps and smartphone applications to provide real-time traffic updates, aiding drivers in navigation.

Putra et al. introduce an Intelligent Traffic Monitoring System (ITMS) framework designed to assess drivers’ punctuality in reaching their destination, even when unexpected events occur [84]. Their ITMS leverages IoT technology to measure speed using four types of sensors: motion, ultrasonic, passive infrared (PIR), and speed sensors. The system includes four monitoring tools: RFID tags for vehicles, roadside sensors, IP addresses for vehicle connectivity, and QR codes, which allow easy scanning by readers installed throughout the city. Sarrab et al. propose an IoT-based system for real-time traffic data collection and processing [85]. This system updates road congestion and accidents through roadside message units, enabling drivers to adjust their routes accordingly. The proposed model uses Wi-Fi to connect roadside sensors with a cloud server, ensuring timely and accurate information dissemination. Also, the authors proposed an algorithm for vehicle detection and physical length estimation based on magnetic sensors, which detect the disturbances in the Earth’s magnetic field caused by moving vehicles. A threshold is applied to identify vehicle presence based on significant magnetic field fluctuations. Two magnetic sensors are placed at a known distance; the vehicle speed ${(v}_{i}$) is calculated by measuring the travel time between two sensors. The vehicle magnetic length ($vml_{i}$) is derived as follows:

(1) $$vml_{i}=v_{i}\cdot ot_{i}$$

where $v_{i}$ is the vehicle speed and $ot_{i}$ the occupancy time. The vehicle’s physical length ($vpl_{i}$) is estimated by subtracting the detection zone length (idz) from the magnetic length:

(2) $$vpl_{i}=vml_{i}-idz$$

with:

(3) $$idz=v_{i}\cdot \left(T_{dep}^{A}-T_{arr}^{A}\right)-d_{A-B}$$

where $T_{arr}^{A}$ and $T_{dep}^{A}$ are the arrival and departure times, whereas $d_{A-B}$ is the distance between the sensor nodes *A* and *B*.

Barbosa et al. introduce an innovative system called LightSpaN, a CNN-based model designed for efficient and rapid training [86]. The system’s performance is assessed using SUMO software and real-world IoT data, achieving a remarkable accuracy rate of 99.9% in identifying emergency vehicles. Their solution improves vehicle detection speed and significantly reduces waiting and travel times. LightSpaN is trained on images from sources such as the COCO API, VOC datasets, Google Images, and IMAGINET.

Kheder et al. [87] propose enhancements to traffic monitoring systems by integrating vehicular cloud computing (VCC) and IoT-assisted robotics (IoRT). Their architecture incorporates IoT sensor nodes and cameras to collect real-time traffic data. Two deep learning models are implemented: a modified version of LeNet-5 for traffic sign recognition and Inception-V3 for detecting traffic lights. Additionally, the authors optimize ultrasonic sensor positioning to prevent accidents. The processed data are transmitted to the cloud and made accessible via a mobile app for drivers and commuters. Results show the LeNet-5 model achieves 99.12% and 99.78% accuracy, while Inception-V3 reaches 98.6%, surpassing other traffic monitoring systems significantly.

Dhingra et al. present an integrated fog and cloud computing framework to address real-time IoT data analytics challenges in urban traffic monitoring and traffic light management [88]. Since traditional cloud computing can suffer from delays due to data overload and network congestion, the authors propose using fog computing nodes (miniature computers) to pre-process real-time data from distributed sensors. This method reduces latency and enhances system responsiveness, leading to more effective management of traffic congestion and accident detection. Additionally, the system connects to Twitter to send congestion alerts, blending technological advancements with practical tools to improve urban mobility. Despite implementing monitoring systems like SAHER in Saudi Arabia, drivers continue to circumvent penalties and violate traffic laws.

**Figure 16:**
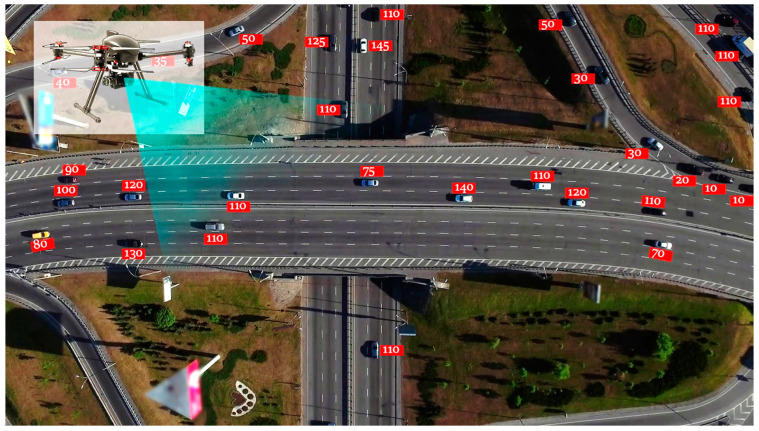
UAV system proposed in [89], able to monitor vehicles’ movement by detecting and highlighting the speeds.

Khan et al. propose an advanced traffic surveillance system leveraging Un-manned Aerial Vehicles (UAVs) and 5G technology to tackle road accidents [89]. The key innovation lies in the combination of UAVs and 5G, offering a more dynamic and responsive solution than traditional traffic monitoring systems. Their results indicate that reducing common traffic violations significantly lowers accident rates, leading to fewer fatalities and injuries. Figure 16 illustrates the UAV system’s monitoring capabilities.

Singh et al. [90] introduce a framework that integrates traffic management with air quality prediction for smart cities using crowdsourced data. Their approach involves creating Navigation Reference Spatial Data (NRSD) derived from GPS and OpenStreetMap trajectories, ensuring spatial reliability. These data feed into a real-time traffic density analysis utilizing k-means clustering and Graham scanning techniques. For air quality assessment, the framework employs a Bayesian classifier based on eight standard parameters. An analysis of three major cities in North India demonstrates 98% accuracy, reflecting a 3.8% improvement over previous models. Additionally, the system suggests optimal routes that avoid traffic in 87% of cases. The framework’s innovation lies in its integrated use of crowdsourced data and advanced spatial–temporal analysis to enhance real-time traffic management and air quality monitoring.

### 4.2. Off-Board Systems for Road and Environmental Monitoring

For road condition monitoring, Åstrand et al. presented a prototype system for road condition monitoring in underground mine tunnels in Sweden [91]. Their proposed system aims to improve production efficiency and reduce vehicle wear. The system consists of three main components. The first is a localizer that uses an extended Rao–Blackwellized particle filter that combines vehicle sensor data with Wi-Fi signal strength from access points. The second is road monitoring using two methods, one based on a Kalman filter in synergy with a vehicle suspension system model and another using road condition measurements based on power spectral density. Finally, re-routing is planned using a rescheduling algorithm to make automatic decisions about roads in poor condition, integrating maintenance activities into the short-term production schedule to minimize operational disruption.

**Figure 17:**
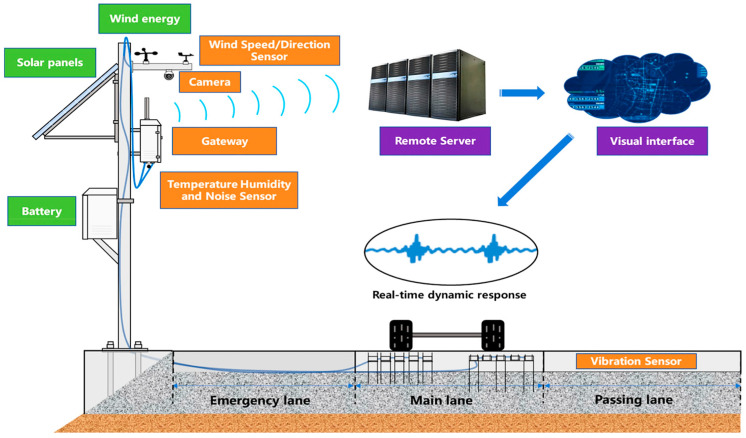
Monitoring system for road pavements using IoT technology proposed in [92].

Ye et al. in [92] developed an intelligent monitoring system for road pavements using IoT technology. The project addresses several challenges related to using sensors for infrastructure monitoring, such as the limited lifespan of sensors, damage caused by sensor insertion, and managing the large amount of data generated. The proposed system includes a sensor network, cloud platform, communication, and autonomous power supply (Figure 17). Specifically, it focuses on cement concrete pavement and integrates sensors with the material structure to collect data on energy, temperature, humidity, noise, wind, and vibration. These data support pavement design and maintenance and promote synergistic applications between vehicles and roads. The authors plan to continue exploring the use of IoT in road maintenance, safety, and material design.

Abdelmalak et al. [93] proposed an innovative method for adaptive and intelligent pothole detection on asphalt, highlighting the importance of integrated approaches for efficient repair of road inspection systems. The method, which integrates advanced software and hardware solutions, has been evaluated by the Pothole Detection Dataset (PDD) and tested by different computer vision models, including VGG19Net, ResNet-50, GoogLeNet and AlexNet. Among them, the most effective was AlexNet, with a 92.15% accuracy, sensitivity of 91.38%, F1 score of 96.52%, and processing time of 279.35 s.

Ye et al. [94] focused on road surface condition monitoring, presenting an IoT-based architecture to enhance service quality and road infrastructure maintenance. They developed a vibration monitoring prototype including wireless sensor nodes, a gateway, a remote server, and a browser interface, and based on multiple layers: data acquisition, pre-processing, processing, interaction, energy management, and networking. The study highlights critical aspects such as data pre-processing, wireless communication, and visualization. The prototype enables the use of IoT-based solutions in traffic and environmental monitoring systems, also improving road maintenance management.

Gaspar et al. [95] addressed the growing demand for safe forest roads, due to increased tourism, climate change, and forest management needs. They proposed enhancing road infrastructure management through systematic data collection and diagnostic activities by designing a temperature profile measurement probe and a simulation model to validate the probe’s effectiveness. This device will be deployed in forest road environments to support ongoing infrastructure monitoring and maintenance.

Hajder et al. [96] developed an automated, maintenance-free system for dosing chemical reagents used in winter road maintenance to combat slipperiness caused by water crystallization, a problem that significantly reduces friction and increases the accident risk. The proposed system collects data during salt spreader operations to determine the optimal reagent density for specific road sections. These data are provided to the thermodynamic models to calculate the ideal reagent concentration and to NNs to detect anomalies. Unlike traditional methods, the goal of the proposed system is to enhance application efficiency, reduce chemical overuse, and minimize the environmental impact.

Chen et al. [97] addressed the challenge of road ice, a major traffic safety concern, by proposing a detection and forecasting system utilizing IoT technology. Their innovation includes a low-power ice sensor that monitors road conditions and transmits data to an IoT gateway via LoRa technology. The system features a distributed algorithm on the gateway that identifies ice formation trends over time, adapting to various road conditions. The algorithm uses road data provided from ice capacitive and temperature sensors, time stamps, and sensors IDs to monitor the icy road’s risks. It switches between two monitoring modes: *Commfreq_Monitor_Model* (low-frequency sampling/transmission) for temperature range [+2 °C ÷ +5 °C] and *Highfreq_Monitor_Model* (high-frequency sampling/transmission) for temperatures below +2 °C. The sensors autonomously switch modes and evaluate icing risks by analyzing temperature trends and sensor data. Additionally, the authors developed a deep NN model called Trans-CGAN to provide accurate ice predictions, even with unbalanced datasets, by combining a time series encoder–decoder structure and generative adversarial networks (GAN)-based classification. Time-series data are processed with position encoding and multi-head attention to extract patterns, while GAN components balance the class distribution by generating positive samples. The proposed multi-head self-attention is a collection of general self-attention mechanisms:

(4) AttentionOutput=Attention(Q,K,V)

where *Q*, *K*, and *V* are the matrices constituted by query, key, and value vectors. This approach improves classification accuracy by leveraging generated and real data to optimize the model performance, thus enhancing the road safety.

Kotus et al. presented a novel method for detecting wet road surfaces using an acoustic vector sensor (AVS) [98]. Their technique analyzes sound intensity in the frequency domain, identifying acoustic events related to vehicle movements. In detail, the following equation was employed to calculate the sound intensity:

(5) $$I\left(\omega \right)=\frac{1}{2\rho r\omega }Im(P_{1}\cdot P_{2}^{*})$$

where ρ is the air density, r the spacing between pressure sensors, and $P_{i}$ the Fourier transform of the pressure $p_{i}$, whereas Im indicates the imaginary parts of a complex spectrum and asterisks, the complex conjugation.

By calculating the sound direction for different spectral components, the system filters out irrelevant noise and estimates the road surface condition from the sound intensity spectrum. In particular, the algorithm identifies acoustic events, such as vehicle sounds, by detecting increases in sound intensity above background noise, determined by an exponential filter with long averaging time:

(6) $$I_{n}\left[k\right]=\alpha {\cdot I}_{n}\left[k-1\right]+(1-\alpha )\cdot I_{n}[k-\delta ]$$

where α is the averaging factor, k the sample index, and δ the update delay.

Afterward, the algorithm evaluates the frequency spectrum of these sounds, with higher intensity in specific high-frequency bands (above 2.5 kHz) serving as a clear indicator of wet road conditions. To ensure reliability, the results are smoothed using filters, reducing noise and accounting for variations in traffic and environmental conditions. In real tests, the algorithm demonstrated an accuracy of about 89% in precision, recall, and F1 score. The study concludes that this technology can be integrated into smart city systems for efficient road water detection, contributing to safer and more responsive urban environments.

### 4.3. Comparative Analysis of Traffic Management and Road Condition Monitoring

The traffic management systems proposed in the analyzed literature have demonstrated excellent performance, utilizing diverse acquisition technologies such as strategically positioned cameras, ultrasonic sensors, RFID tags, and magnetic sensors. A critical feature of any effective traffic management model is a seamless interconnection between vehicles, infrastructure, and users, which helps reduce congestion by providing drivers with real-time information and suggesting alternative routes to avoid traffic. Such real-time updates are particularly beneficial for urban and extra-urban road networks, enhancing overall mobility and driver awareness. An intriguing aspect of the model discussed in [25] is its dynamic traffic light management capability, which improves traffic flow and prevents queue formation. Effective control of traffic lights within urban centers is essential for smooth traffic operations and minimizing delays. Another significant advantage of these systems is their ability to monitor traffic violations (speed limit breaches, dangerous driving, and other infractions) through strategically placed cameras and sensors, fostering greater driver responsibility and road safety by reducing accidents. In this regard, Section 5.3 provides an overview of various commercial hardware and software solutions for traffic control and management. Table 9 summarizes the key common features of the systems proposed in the articles reviewed in Section 4.1.

Monitoring road surface conditions is equally important. Cameras and sensors properly placed can assess road wear and facilitate predictive maintenance, ensuring safer driving conditions and extending the road lifespan. Table 10 compares the proposed systems based on performance metrics, communication technologies, involved entities, and detection sensors, offering a comprehensive evaluation of their capabilities.

Table 9 and Table 10 highlight that not all the articles analyzed consider the critical role of communication between vehicles and infrastructure (V2I), which is essential in the context of smart cities. First, V2I improves traffic management by enabling better coordination of traffic lights and faster adaptation to real-time road conditions; it also allows the reduction of waiting times, alleviation of congestion, and improvement of vehicle energy efficiency, leading to lower CO_2_ emissions. Second, V2I enhances road safety by enabling vital information exchange, such as weather alerts or accident reports, allowing drivers to adjust their behavior and avoid potential accidents. Additionally, V2I is key to integrating autonomous vehicles with smart infrastructures into urban environments, enabling route optimization and better interoperability, making cities more accessible, and reducing parking needs. In conclusion, V2I is a technological advancement essential in transforming cities into smarter, more livable places that can address mobility and environmental challenges.

Current technologies for traffic monitoring and management, although advanced, have some limitations that hinder their effectiveness, concerning technological aspects related to data accuracy, limited coverage on the road network, and fragmented data caused by discontinuous communication between different monitoring platforms. The growing amount of data coming from sensors, connected vehicles, and mobile devices requires high computing power and sophisticated algorithms to support real-time analysis; furthermore, the poor quality of data (noisy or incomplete data) can lead to errors and ineffective decision management. Moreover, connected monitoring systems can be vulnerable to cyber-attacks, making data privacy an ongoing challenge. The enhancement of infrastructures with advanced sensors (better performance and resistance to environmental conditions), the big data techniques, predictive analytics, and the adoption of digital twin models are emerging technologies that offer new opportunities for traffic monitoring and management. A further challenge is that non-centralized and distributed systems may not be able to determine alternative routes based on information about the level of traffic.

## 5. Cutting-Edge Commercial Solutions for Monitoring Vehicle, Driver, and Traffic Conditions

This section describes the most recent commercial hardware and software solutions in the field of advanced driver and vehicle monitoring systems, as well as out-of-vehicle solutions for monitoring traffic, environmental, and road conditions.

### 5.1. Overview of Commercial Solutions for Driver Condition Monitoring

**Figure 18:**
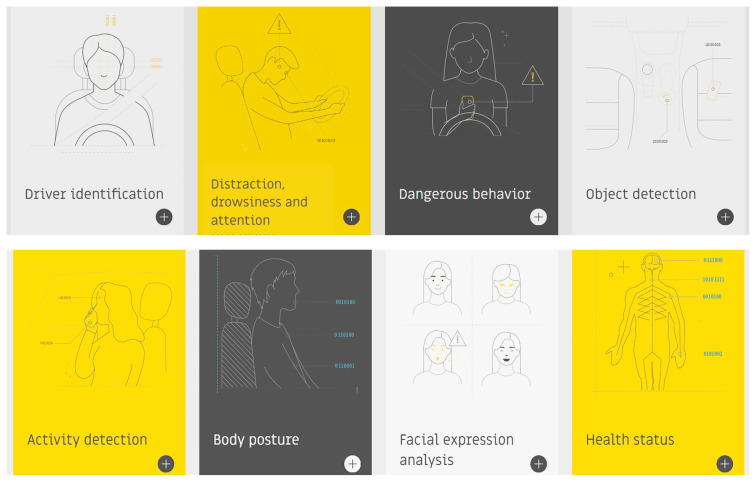
Key features of the “Smart Eye Pro 12” DMS released in 2024 by Smart Eye company [99].

Many companies are focusing on designing a DMS based on cameras or sensors to monitor driver behavior and alertness. By processing in-cockpit data in real time through advanced algorithms, the DMS detects signs of distraction or drowsiness (main causes of accidents), preserving driver privacy by processing data locally. In May 2024, Smart Eye Co. (Göteborg, Sweden) introduced the Smart Eye Pro 12 eye-tracking system, featuring Profile ID and advanced drowsiness detection capabilities to enhance safety and the user experience (Figure 18) [99]. The Profile ID function assigns each user a unique identifier based on facial features, removing the need for manual identification. The drowsiness detection technology uses algorithms to assess it on a scale from 4 to 9, in line with upcoming 2026 regulations mandating such features for vehicle safety. The Smart Eye Pro 12 release also includes gaze accuracy and pupil tracking for heightened robustness and reliability. The system features a multi-camera setup, with two to eight cameras to be placed horizontally or vertically, so enabling 360-degree head and eye tracking. AI-based emotion software developed by Affectiva Co. (Boston, MA, USA) allows the Smart Eye’s DMS to capture a variety of features in real time [99]:

1. Driver identification: it recognizes the driver to adjust the vehicle’s settings accordingly and ensures that only authorized drivers can use the car.
2. Distraction, drowsiness, and attention: it monitors the driver’s eye, head, and facial movements to detect distraction and catch early signs of fatigue, ensuring attention remains on the road.
3. Dangerous behavior: it identifies distracting behaviors such as eating, drinking, smoking, or using a mobile phone.
4. Object detection: it detects objects within the vehicle and monitors how the driver interacts with them.
5. Activity detection: it monitors the driver’s activities to determine if they are focused on driving or engaged in other tasks.
6. Body posture: it monitors the driver’s posture, movements, and interactions with objects or vehicle interfaces.
7. Facial expression analysis: Affectiva’s Emotion AI interprets the driver’s facial expressions to assess his/her mood, emotions, and behaviors.
8. Health status: it assesses the driver’s health status by analyzing body posture along with eye, head, and facial movements.

**Figure 19:**
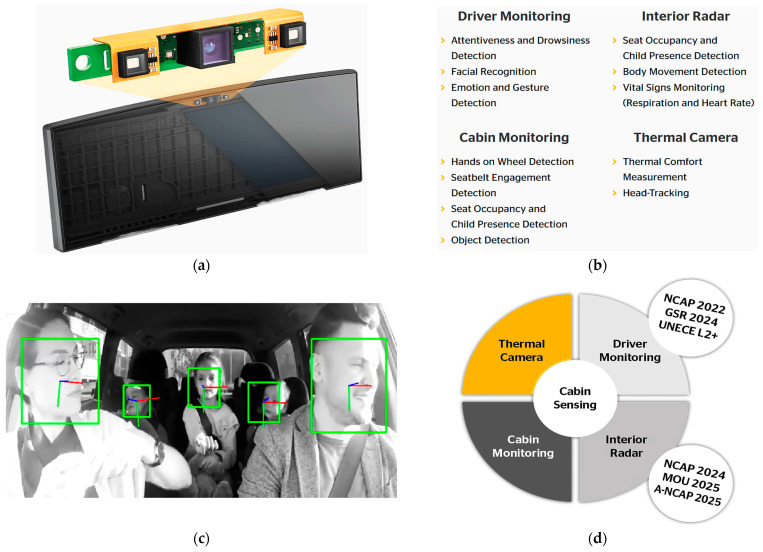
(**a**) Interior camera system enabling driver and vehicle to interact seamlessly; (**b**) developed technologies and detection capabilities from Continental Engineering; (**c**) hardware and software solutions for face gesture detection; (**d**) key features of “Cabin Sensing” solution and standards met [100].

Recently the Continental Engineering Co. (Frankfurt, Germany) proposed the “Cabin Sensing Solution” with technologies and detection capabilities, including a DMS, radar, cockpit monitoring system, and thermal camera (Figure 19a,b,d) [100]. The DMS detects the driver’s drowsiness and distraction, and supports facial recognition, emotion detection, and advanced controls (Figure 19c). Its main component is the DMS software algorithm, which operates on dedicated hardware by a CAN-FD interface or is integrated into a third-party High-Performance Computer (HPC). A 1MP camera with LED illumination is available as an encapsulated unit or integrated within the instrument cluster [100].

The interior radar enables the child presence detection (CPD) in compliance with the EuroNCAP 2024 standard (more details at the website https://cdn.euroncap.com/en/ (accessed on 16 December 2024)). In addition, it tracks body movements and vital signs, including RR and HR, to detect seat occupancy and any children [100]. The monitoring system covers the driver, all passengers, and the full cabin, detecting the presence of passengers and objects, seat occupancy and seatbelt use, and assessing the occupants’ posture and status for Level 4 and 5 vehicles. For example, it checks if the driver is focused and their hands are on the wheel. Also, the system includes driver identification (Driver ID) for personalization and support for video conferencing [100]. The thermal camera measures the temperature of the head, forehead, and body, calculating a Thermal Comfort Value (TCV) for both driver and passengers. This numeric scale reflects their thermal sensation, from very cold to very hot, and can be valuable input for optimizing climate control [100].

**Figure 20:**
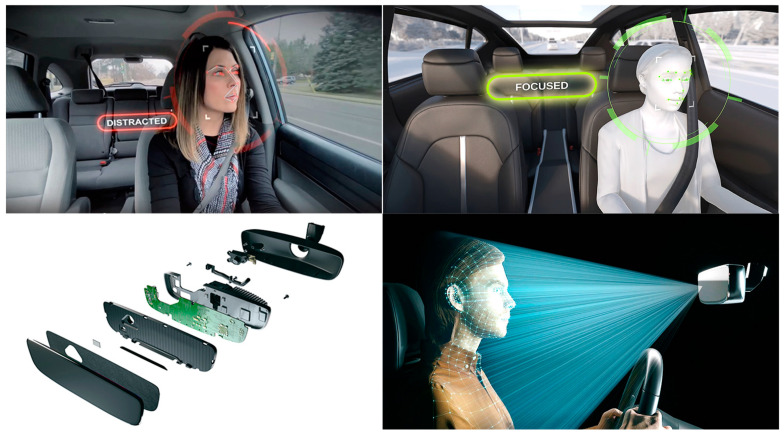
Key features of Magna DMS: distracted driver detection, drowsy detection, occupant detection, child presence/seat detection, occupant classification, properly worn seatbelt detection.

Magna International Inc. (Aurora, ON, Canada) recently launched a Driver and Occupant Monitoring System (DOMS) that uses ADAS cameras and interior mirrors to detect driver distraction, drowsiness, and fatigue, aiming to reduce driving distractions and thus crash risks (Figure 20). The Magna DOMS, integrated into the rearview mirror, analyses the driver’s head, eye, and body movements to detect signs of distraction or fatigue. By using cameras and infrared sensors, it can detect the loss of concentration of the driver, warning him by visual or audible alerts [101]. In particular, the proposed technology is able to distinguish between routine driving actions, such as checking the side mirrors, and real distraction actions; once the system recognizes them, it alerts the driver with acoustic or visual signals. A camera in the interior rearview mirror allows monitoring of the driver but also other occupants. This system can include features like child presence detection, seatbelt use verification, and passenger identification, enabling personalized settings based on user preferences [101]. Infrared ethanol sensors positioned near the driver monitor the alcohol and CO_2_ levels in the driver’s diluted exhalations to determine any BAC level above the legal limit. Magna meets the Euro NCAP and General Safety Regulation requirements [101].

**Figure 21:**
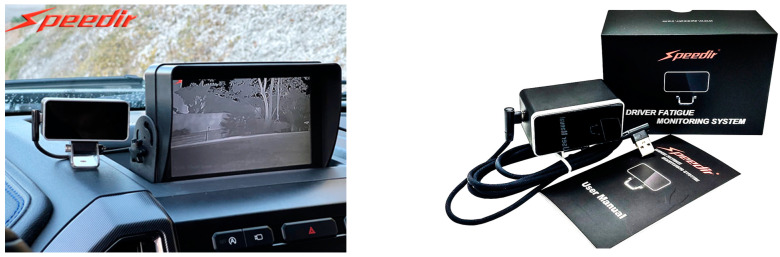
Driver Fatigue Monitoring System for driver’s safety by Speedir [102].

The last system, proposed by Speedir Inc. (Santee, CA, USA), is an AI dash cam with eye-tracking functionality to detect drowsy driving. It currently operates as a standalone tool and does not integrate with typical fleet management platforms. The Driver Fatigue Monitoring System (DFMS) features a solution for preventing drowsy driving [102]; equipped with a night vision camera and AI facial recognition algorithms, this system tracks signs of drowsiness and distraction using infrared sensors to monitor the eye positioning (blink rate, closed eyes, eye direction), head movements (e.g., nodding off), and yawning, even when the driver is in low light or wearing glasses. Speedir’s DMS is powered by pre-trained AI software, requiring no Wi-Fi for operation or updates (Figure 21). This system enhances driver safety by alerting users to fatigue or distraction. The key features of the system are as follows:

- Utilizing AI algorithms, Speedir’s DMS detects drowsy or distracted driving by tracking eye and head movements, issuing audible alerts for cell phone use, falling asleep, or other distractions.
- With infrared technology, the system performs day or night, unaffected by reflective light or any glasses the driver may wear.
- The DMS is designed for universal plug-and-play USB installation, fitting seamlessly in any vehicle.
- Privacy is protected as it operates without internet or cloud connectivity.

Table 11 compares the commercial DMSs previously analyzed, considering their detection capabilities, integrated sensing devices, monitored human body and car parts, and developed detection algorithms, offering a comprehensive evaluation of potentialities.

### 5.2. Overview of Commercial Solutions for Monitoring Vehicle Conditions

An In-Vehicle Monitoring System (IVMS) consists of one or more electronic devices and purpose-designed software installed in a vehicle to capture and record information about vehicle performance and driver behavior. Often referred to as the “black box” of the automotive world, an IVMS typically logs live data such as speed, acceleration, braking patterns, seatbelt usage, and more. These data provide a picture of activities across a single vehicle or fleet of vehicles. Moreover, a robust IVMS evaluates driver and vehicle performance against pre-defined safety standards and criteria. IVMSs range in complexity: some connect via the vehicle’s OBD-II port for easy plug-and-play functionality, while others require installation and enable real-time tracking and data downloads. IVMSs offer a wide range of advantages that enhance the safety, efficiency of vehicles, and cost management in the case of the fleet of vehicles:

- Enhanced driver safety: improve safety by providing feedback on unsafe driving behaviors.
- Improved driving behavior: discourage unsafe actions like speeding, harsh acceleration, or abrupt braking.
- Crash detection and response: this allows identification and a quick reaction to accidents, ensuring timely assistance to distressed drivers.
- Vehicle performance tracking: monitor fuel consumption, mileage, engine health, and other crucial factors to identify potential issues before they become costly problems, extending the lifespan of vehicles.
- Compliance with health and safety standards: help employers meet legal obligations by monitoring driver hours and addressing fatigue, demonstrating a commitment to safety and duty of care.
- Fewer incidents and crashes: reduce accidents by raising awareness of risk factors and improving driving habits.
- Lower insurance premium: offer detailed insights into incidents, enabling efficient claims handling and accurate allocation of responsibility (many insurers provide premium discounts for fleets using IVMS).
- Enhanced load and vehicle security: help protect cargo and vehicles from theft or tampering by tracking their locations in real time.
- Reduced fuel costs and environmental impact: lower fuel expenses and maintenance costs by providing insights into fuel consumption, promoting efficient routing, and reducing idling.
- Live GPS tracking and geo-fencing: GPS tracking provides an overview of vehicle locations, and, at the same time, geo-fencing enables the setting of boundaries for vehicle usage, such as speed limits in specific areas or restricting access to certain zones.

**Figure 22:**
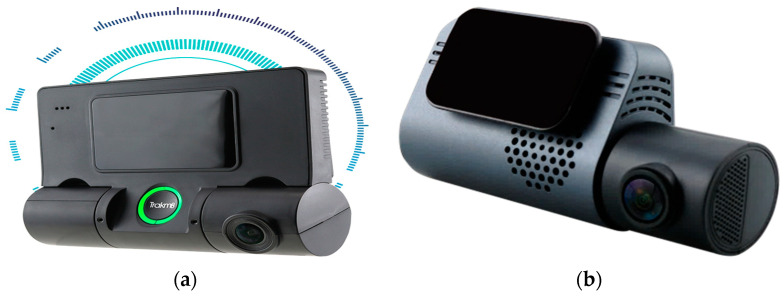

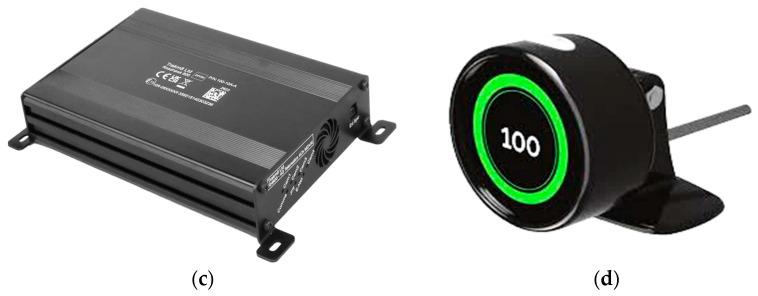
Solution for vehicle condition monitoring by Trakm8: (**a**) 4G Integrated Telematics Camera RH600 [103]; (**b**) RoadHawk DC-4 Dash Cam [104], (**c**) RH800 4G Mobile Digital Video Recorder [105]; (**d**) ACC750 Driver ID and Feedback device [106].

Many companies are focusing on developing new IVMSs based on radar, cameras, GPS, or multi-sensor setups. In the following section, some cutting-edge commercial solutions are reported. Trakm8 is a technology company (Birmingham, UK) focused on automotive telematics solutions and connected vehicles, including fleet cameras, multi-camera systems, dash cams, and related software. The 4G RH600 camera offers telematics capabilities, including driver behavior scoring, vehicle health alerts, driver ID, and upcoming ADAS features to enhance road safety and reduce risks (Figure 22a). Connectivity technical specifications are Bluetooth Low Energy (BLE 4.2), GPS, 4G technology, vehicle CAN 1, and Tacho CAN 2 interfaces [103]. The RH600 camera (manufactured by Trakm8 Holdings PLC, Birmingham, UK) can be used as a traditional dash cam in two different ways, with a single lens facing the road/driver or a dual lens facing both the road and driver. The RH600 features the Trakm8’s ConnectedCare vehicle health software solution, designed to reduce breakdowns and non-start risks, manage service and repair costs, and enhance residual vehicle value. Trakm8 Insight is a software platform that processes data from the RH600 camera and displays it via a desktop portal or mobile app. The RH600 camera will implement ADAS features, including driver distraction and fatigue detection, forward collision warnings, and following distance warnings.

Trakm8’s technology also integrates the RoadHawk DC-4 forward-facing dash cam (Figure 22b), equipped with Wi-Fi, GPS, and a 1440p Quad HD (2K) camera (30 fps frame rate), which can capture road signs and license plate numbers, so allowing drivers to save photos and videos via a gesture sensor. Moreover, it also features a built-in G-Sensor that automatically allows drivers to save road accident video for later review [104]. The systems designed by Trakm8 also include the RH800 4G Mobile Digital Video Recorder (MDVR), which supports up to four cameras and features telematics functionality, including GPS tracking, driver behavior, and vehicle health monitoring (Figure 22c) [105]. The Trakm8 Insight telematics system can measure speed, over-revving, and harsh acceleration and braking. Trakm8 Insight telematic utilizes GPS, fuel consumption, driving trends, and dash cam data to offer information supporting management processes.

Trakm8’s ConnectedCare vehicle health solution provides diagnostic data by accessing the CANbus network of cars and commercial vehicles. This system identifies Diagnostic Trouble Codes (DTCs) or engine fault codes characteristic of specific vehicle issues and displays them on the Insight software platform. The Insight platform allows remote monitoring of different vehicle warning lights, such as washer fluid level, ABS, traction control, Diesel Particulate Filter (DPF), AdBlue levels, and tire pressure, and it alerts vehicle owners and fleet managers to potential issues early for proactive maintenance.

Trakm8’s Driver ID authentication software allows drivers to log into their assigned vehicles using standard door entry cards, employee ID cards, or NFC-enabled mobile devices. Drivers can utilize the ACC750 Driver ID, a combined driver identification and feedback solution connected to different Trakm8 telematics solutions via Bluetooth or a wired connection (Figure 22d) [106]. The ACC750 features a multi-function button that allows drivers to switch between business and private travel modes. Holding the button for three seconds triggers an alert sent to the paired telematics device, which then relays the message to the server for monitoring through a web portal or mobile app.

**Figure 23:**
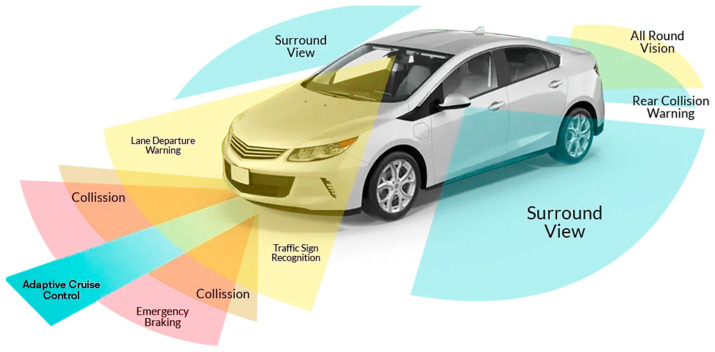
ADAS functionalities by TrackoBit devices: forward, rear, or side collision alerts, signal violation, lane switch alert, and over-speeding alert.

Another leading IVMS available on the market is the TrackoBit GPS tracking software proposed recently by InsightGeeks Solutions Pvt. Ltd (Noida, India), a mobility technology company [107]. TrackoBit software is compatible with a lot of GPS hardware and IoT devices, making it compatible with various manufacturers; its key features are the driver behavior management and monitoring, ADAS interface, video telematics with geospatial tracking solutions, and route planning and management. Driver behavior management and monitoring is based on a GPS tracking system and various sensors, providing data on driver performance, distinguishing between high- and under-performing drivers, and verifying if they frequently deviate from the assigned route. This software optimizes route planning to maximize operational efficiency and collects, displays, and analyzes data related to sharp cornering, harsh acceleration and braking, and over-speeding. The TrackoBit ADAS provides views of the vehicle from all angles and is notified whenever the system recognizes any anomaly or dangerous situation (Figure 23).

**Figure 24:**
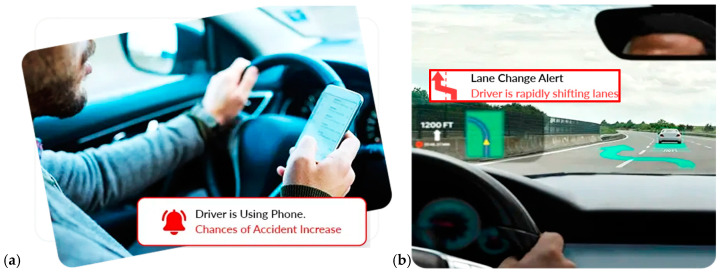
Video Telematics by TrackoBit system: DMS alert example (**a**), ADAS alert example (**b**).

**Figure 25:**
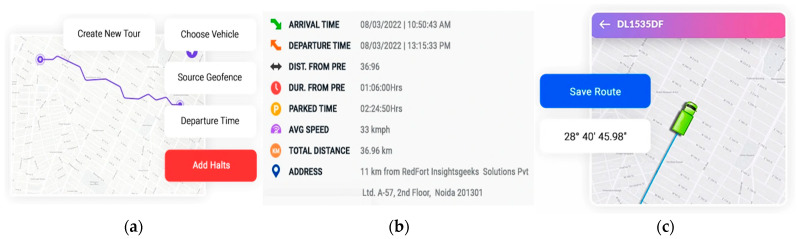
Route planning and management software by TrackoBit: (**a**) create tour, (**b**) monitor route, (**c**) manage trips.

An AI-powered DMS and ADAS constitute the two pillars of Video Telematics by TrackoBit system, which is integrated into vehicles via the OBD ports (Figure 24). Route Planning and Management Software by TrackoBit offers trip planning and management capabilities based on three steps (Figure 25). The first step, create tour, assists in designing a personalized tour by entering the desired delivery location, setting the total trip time, and arranging the designated stops. The second step, monitor route, assists in gaining real-time visibility into vehicle locations and movements, monitoring their progress along specified routes, and saving optimal paths for repeated journeys. The third step, manage trips, assists in utilizing live tracking to oversee trips while vehicles are in transit.

**Figure 26:**
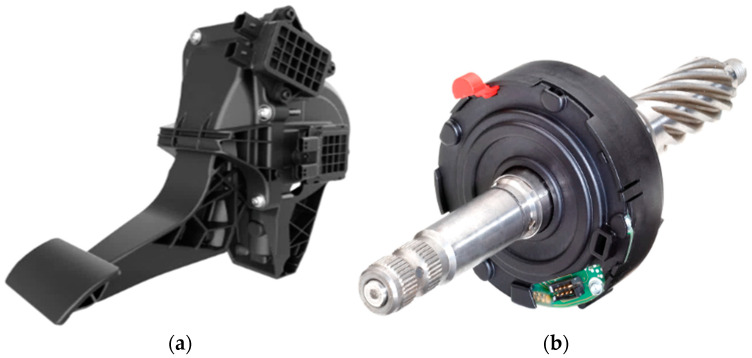
By-wire technology by FORVIA: brake-by-wire system (**a**) and steering torque sensor (**b**).

Another interesting IVMS available on the market is the recently proposed technological system by the automotive group FORVIA (Nanterre, France), an automotive group that develops technologies to be installed inside or outside vehicles to support the decision making of drivers and automated systems. The IVMS includes three technologies: by-wire technology, radars and environmental sensors, and a vision system; the first includes a brake pedal sensor (BPS) (Figure 26a), simulating the feeling of a conventional brake system while electronically transmitting driver input. The BPS transfers the driver’s action to the brake control unit, which provides a signal to the braking system [108]. The by-wire technology also proposes a steering torque sensor; as the driver turns the steering wheel, the sensor measures the torque applied to the steering column. The sensor detects the torsion bar angle and provides information to the control module, which calculates the necessary servo assistance to support the driver’s action [109,110].

**Figure 27:**
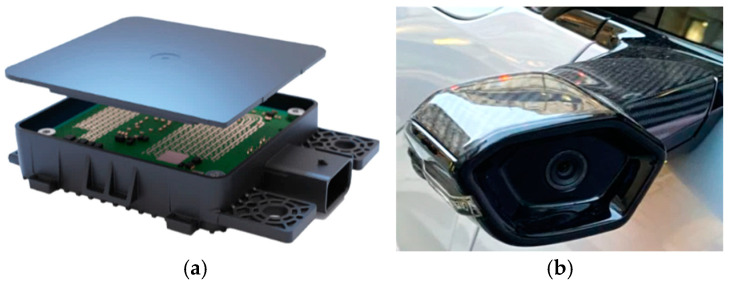

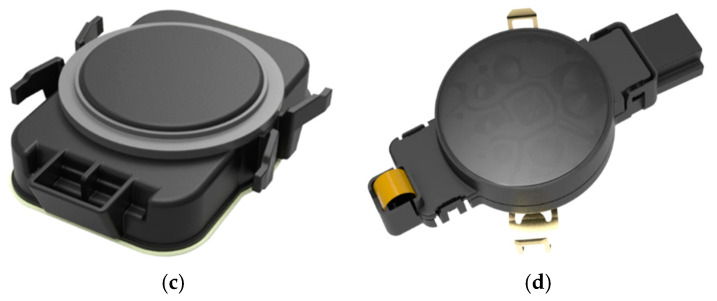
Radars and environment sensors by FORVIA: 77GHz radar (**a**), e-Mirror camera (**b**), SHAKE road condition sensor (**c**), HELLA Rain Light Sensor (**d**).

Radars and environment sensors by FORVIA include a 77GHZ radar, an eMirrors camera, a road condition sensor, a rain-light sensor, and a parking system (manufactured by FORVIA company, Nanterre, France) (Figure 27). The radar technology supports ADASs, enhancing environmental detection by identifying stationary elements like road boundaries and tracking dynamic objects such as pedestrians, cyclists, and vehicles [111]. The eMirrors camera replaces traditional external mirrors, providing a surrounding view; the lightweight and aerodynamic design reduces CO_2_ emissions and improves energy efficiency. The road condition sensor detects water on the road in real time, estimating the grip by vibration measurement through a piezoelectric sensor [112]. The rain–light sensor is a multi-function device with the following capabilities: rain detection for automatic wiper control, light sensing for automatic headlight activation and tunnel or overpass detection, sun load measurement, head-up display brightness adjustment, and humidity/temperature sensing to prevent windshield fogging [113].

**Figure 28:**
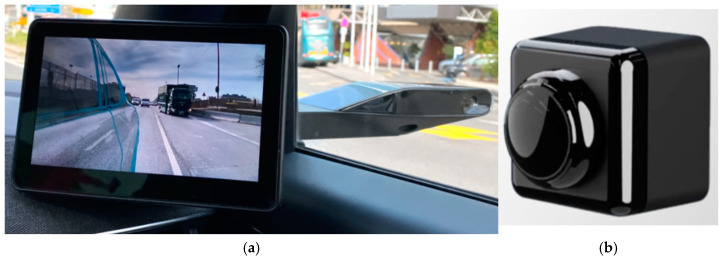
Vision systems by FORVIA: eMirror UX Safe engine (**a**), surround view system (**b**).

Vision systems by FORVIA include an eMirror UX Safe engine and a surround view system [114]. The eMirror UX Safe engine is a software that enhances mobility safety by using a camera-based system to improve drivers’ visibility and situational awareness (Figure 28a). It provides safety alerts, optimizes energy efficiency, and minimizes distractions, ensuring clearer views even in challenging environments and lighting conditions. The Vision system’s surround view enables a 360° panoramic perspective of the car using a four-camera fisheye setup (Figure 28b). The surround view system has been conceived to enhance safety, have better visibility, and effortlessly maneuver. Combining data from these cameras recreates the entire environment and offers features such as virtual transparency with gaze detection.

**Figure 29:**
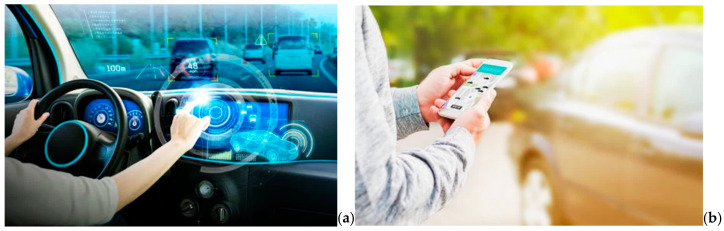
(**a**) The vehicle learns the driving style from the driver, to be integrated with preloaded programs and duplicated when same conditions occur. (**b**) Sharing data with other users via web platforms.

The last IVMS described in this section, although not yet on the market, is particularly interesting for understanding future trends; it is a system developed by Social Self Driving S.r.L (Como, Italy) that captures and replicates a driver’s unique driving style in autonomous vehicles. This technology allows driverless cars to mimic the behavior of their owners or professional drivers, selecting from a range of pre-recorded profiles [115]. The system utilizes hardware and software components commonly found in modern vehicles with assisted or autonomous driving capabilities. It integrates various sensors, including those that measure steering angles, torque, and speed, as well as sensors for accelerator and brake pedal actions, yaw, pitch, roll, and both lateral and longitudinal acceleration. From these data, the system profiles a driver’s style, which can be set or shared through a dedicated cloud platform, allowing it to be programmed into autonomous or semi-autonomous vehicles (Figure 29). The “Social Self Driving” app allows users to access profiles made by others, creating a virtual marketplace where car manufacturers, professional drivers, driving instructors, and public figures can offer and sell customized driving programs [115].

Table 12 compares the IVMSs analyzed in Section 5.2, considering the applications, integrated electronic devices and sensors, monitored vehicular parameters, available connectivity technologies and interfaces, and developed software to offer a comprehensive evaluation of their capabilities.

### 5.3. Hardware and Software Commercial Solutions for Environmental and Traffic Monitoring

Traffic monitoring and management services can significantly enhance urban mobility by reducing accidents and emissions. These services, based on seamless communication between infrastructure, vehicles, and drivers, suggest alternative routes (if necessary) via road alerts or real-time in-car messages. Various companies have introduced specialized hardware and software solutions for traffic monitoring and driver assistance.

**Figure 30:**
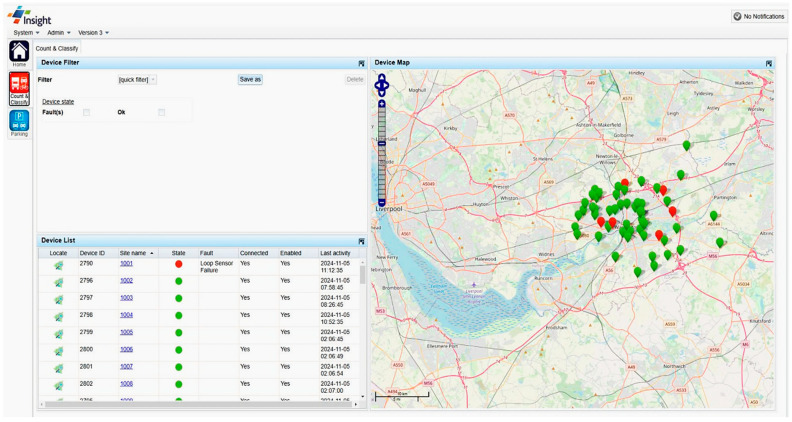
Insight Count and Classify software interface developed by Clearview Intelligence [116].

ClearView Intelligence (Milton Keynes, UK), a company specialized in mobility solutions and data analytics, provides hardware and software for traffic monitoring, mobility management, and predictive analytics. The AI-driven Insight Count and Classify software monitors road traffic, counting and categorizing vehicles at key points, such as intersections or highway segments [116]. Figure 30 illustrates its user interface, representing a solution for enhancing travel safety and efficiency. The operating principle of Insight Count and Classify software is based on some key features:

- Data collection: the software uses devices (such as cameras or sensors) in different environmental conditions to collect information about passing vehicles and traffic.
- Visual analysis: the software analyzes images of vehicles to identify their characteristics, such as type (car, truck, motorcycle, etc.), size, and speed.
- Data processing: the data are processed through algorithms that allow vehicles to be counted and classified in real time.
- Reporting and visualization: the analysis results are presented in reports and dashboards, providing information on traffic patterns, congestion peaks, and other metrics useful for traffic planning and management.

**Figure 31:**
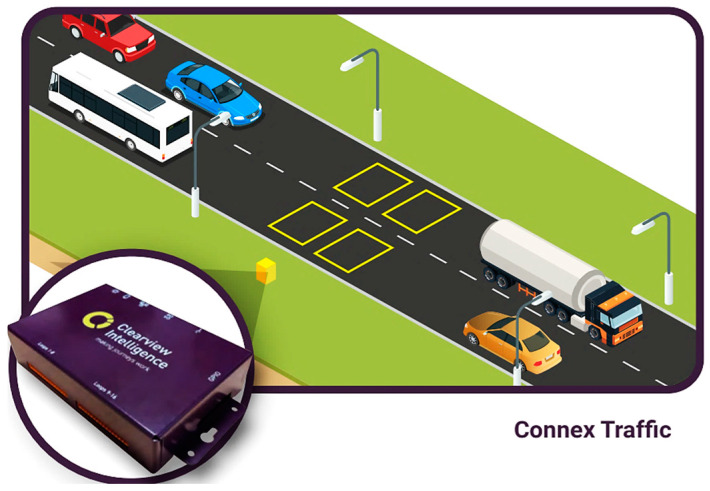
Connex Traffic device is installed on the road: a typical scenario [117].

The Insight Count and Classify software targets stakeholders, including transport and urban authorities, infrastructure project developers and mobility service providers, to deal with traffic management, develop road infrastructure, and optimize vehicular services. Clearview Intelligence also offers hardware solutions for traffic management. Connex Traffic is a technology designed to tackle traffic management and road safety through data utilization and analytics [117]. It employs a network of connected sensors and devices to collect real-time traffic data (including vehicle flows, speeds, and road conditions) from cameras, induction sensors, and GPS devices (Figure 31). These data are processed using algorithms and AI techniques to identify traffic patterns, detect congestion, and assess driving behavior. The analyzed information helps optimize traffic signals, improve road signage, and develop strategies to reduce congestion. Real-time updates can also be provided to drivers via apps or information panels.

**Figure 32:**
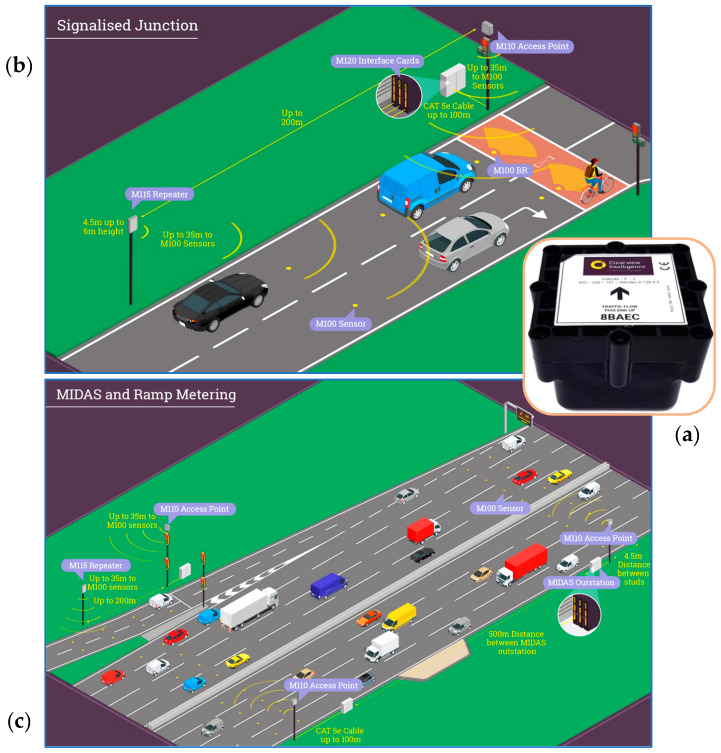
M100 system developed by Clearview Intelligence (**a**), application scenarios (**b**,**c**) [118].

The M100 system by Clearview Intelligence is designed to be concerned with the management and efficiency of transportation and urban mobility infrastructure (Figure 32). It includes sensors, cameras, radar, and other IoT devices to gather real-time data on traffic flow, road user behavior, and environmental conditions [118]. These data are processed using AI and ML algorithms to detect patterns, identify accidents, and monitor congestion. By combining historical and real-time data, the system can predict traffic flow and suggest strategies to optimize traffic and minimize waiting times. The M100 system can also be integrated with other infrastructure and traffic management systems, facilitating communication and coordination across different entities.

**Figure 33:**
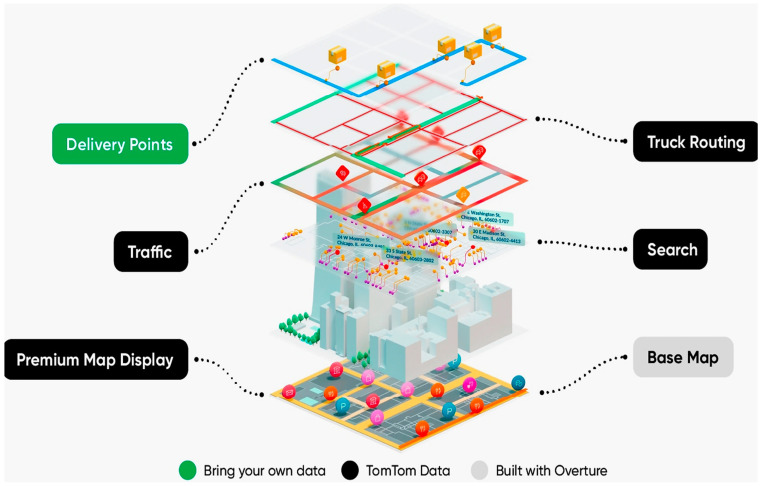
Different layers of TomTom’s Orbi Maps software.

TomTom (Amsterdam, the Netherlands), a company specialized in navigation software and applications, is known for its GPS navigation products and mapping solutions. One of its latest solutions is TomTom Orbis Maps [119]. This mapping software delivers maps and navigation data customized for various devices and applications. Orbis Maps (Figure 33) integrates driver data with information from sources like Overture Maps Foundation™ and OpenStreetMap, ensuring data acquisition at multiple levels. The software combines geographic data from different sources, including satellite imagery, road surveys, and local insights, to produce accurate and detailed maps. By combining these mapping data with GPS technology, TomTom aims to provide efficient and reliable navigation for users.

TomTom offers map updates that incorporate road changes, new constructions, and real-time traffic data. Applications built on Orbis Maps feature an intuitive user interface, making it easy to search for destinations, plan routes, and access information about live traffic conditions and points of interest. Beyond standard road navigation, Orbis Maps offers functionalities for various needs, including commercial vehicle route planning, cycling, and pedestrian navigation, with data fitted to the user. Some TomTom services are cloud-based, enabling access to maps and data across multiple devices. TomTom Orbis Maps provides geolocation tools for companies involved in fleet management, delivery route optimization, and other location-dependent operations.

The main differences that emerge between the Insight Count and Classify software and Orbis Maps are the following:

- Intended use: the Insight Count and Classify software focuses on data collection and analysis for traffic monitoring and management, whereas TomTom Orbis Maps provides detailed digital maps for a variety of applications, including navigation and vehicle automation;
- Technology: Insight Count and Classify uses physical sensors to collect data in the field, whilst Orbis Maps combines data from various sources, including open-source information and vehicle observations to create up-to-date digital maps.
- Target users: the Insight Count and Classify software is faced to road authorities and city planners who need accurate traffic data, whereas Orbis Maps serves a broader range of industries, including automotive and logistics, that require advanced digital maps for a variety of applications.

In summary, both software operate in the traffic management and mapping domain; Insight Count and Classify is specialized in traffic data collection and analysis, whereas Orbis Maps is a flexible and detailed digital mapping platform for industrial applications.

## 6. Challenges and Future Perspectives

The growing number of vehicles on the road have led to a significant increase in accidents and frequent traffic congestion, often bringing cities to a standstill. This challenge has driven research toward developing innovative solutions to improve road traffic through driver assistance architectures. The goal is to reduce accidents by creating advanced driver and vehicle monitoring systems while developing a communication network between vehicles and infrastructure. Moreover, these innovative systems aim to optimize traffic flow by offering drivers alternative routes to their destinations. Concerning driver monitoring, low-intrusive systems, such as sensors integrated into the vehicle interior and cameras, are identified as the most effective solutions. These systems maintain high driving comfort while accurately detecting biophysical parameters to identify health anomalies, drowsiness, stress, and intoxication. Similarly, the use of cameras for driver monitoring produces excellent results but raises issues regarding data security and privacy, since sensitive data are collected such as facial expressions, biometric details, and eye movements; therefore, these non-intrusive solutions pave the way for the implementation of more advanced data security techniques to prevent unauthorized access and tampering, according to the main regulations (GPDR and CCPA). These challenges can be addressed by employing end-to-end encryption techniques, localized processing, and data anonymization or pseudo-anonymization techniques. Also, network technologies present significant challenges, the main one being the latency reduction that allows them to provide real-time feedback and overcome limitations related to limited bandwidth and congested communication channels. The solutions to these problems lie in the use of advanced communication systems (5G-6G), and where possible, in the exploitation of edge computing technologies. Another aspect is the economic one, related to the investment costs for the acquisition of such technology, its maintenance, and upgrading. Scalable designs, economies of scale, and possible government incentives could allow wider adoption of these systems in next-generation vehicles. Another issue concerns the precision and reliability of the proposed systems in detecting the driver and vehicle parameters; since they are continuously subjected to vibrations and mechanical stresses, their accuracy could be compromised. For this purpose, solutions based on advanced AI models, advanced sensor systems, and continuous learning could guarantee high performance for the DMS.

In the field of vehicle condition monitoring and driver behavior detection, a recurring approach in the reviewed literature considers the vehicle’s OBD-II system. By integrating OBD-II data with Machine Learning (ML) algorithms, these solutions enable comprehensive monitoring and predictive maintenance, including vehicle stability, emissions levels, tire health, and driving style and behavior analysis. Finally, Vehicle-to-Infrastructure (V2I) communication is highlighted as a critical component of traffic and road management. V2I enables a continuous exchange of data useful for optimizing road signage, providing alternative routes, and allowing constant road condition monitoring. Emerging technologies such as 6G communications and blockchain promise to significantly improve the efficiency, safety, and reliability of Vehicle to Everything (V2X) systems. In particular, 6G technology enables low latency, high reliability and data rate, and advanced connectivity, allowing better traffic management and efficient vehicle coordination. Blockchain technology offers decentralized and tamper-resistant data management, addressing several challenges in V2X systems. It enables data security and integrity, availability of a decentralized public key infrastructure (DPKI), and transparent and immutable ledgers. In November 2024, the U.S. Federal Communications Commission approved new rules to promote the use of cellular V2X (C-V2X) communication technologies. This initiative aims to improve road safety by enabling vehicles to communicate with other vehicles, infrastructure, cyclists, and pedestrians, thereby facilitating warnings of hazardous conditions such as speeding vehicles, weather conditions, or traffic congestion. Such capabilities enable targeted maintenance efforts, ensuring greater safety and comfort for road users.

Future efforts should focus on advancing AI applications for driver monitoring and the analysis of safety-critical events. A key development is integrating DMS data with road scene analysis using AI techniques to enable early real-time crash prediction. A mobility management system (MMS) can be developed based on intelligent vehicles able to monitor vehicle and driver health, creating an interconnected network by exchanging information in real time with on-the-road infrastructures, and informing users about traffic, crashes, and environmental conditions. Each vehicle exchanges data via V2X technology with surrounding ones and road infrastructure to integrate information about the driver’s status with that provided by nearby vehicles and the road infrastructure. The multimodal analysis of such information, based on DL algorithms, could improve the determination of the driver’s risk level, contextualizing it to the scenario in which the vehicle is traveling.

Moreover, several strategies should be pursued to promote greater transparency regarding AI implementation in DMS and risk events analysis. These should include establishing industry standards, fostering research collaboration, and developing and implementing open-source platforms. Driver Monitoring Systems (DMSs) are becoming a key feature in modern vehicles, especially as we move toward advanced driver assistance systems (ADASs) and even autonomous driving. To ensure these systems work effectively across different vehicles and regions, standardization is crucial. Organizations like UNECE, Euro NCAP, and NHTSA are laying the groundwork for what a DMS should do; in detail, a standardized DMS should monitor and detect driver attention and health, fatigue, and driving style. For example, from July 2024, the European Union has made it compulsory to install alcohol detection systems in new vehicles, which communicate with the electronic control unit to prevent the engine from starting when the driver is under the influence of alcohol. One of the big challenges is ensuring that all the different pieces—cameras, sensors, and software—can work across brands and models. Communication protocol to support the data exchange between vehicles and infrastructures needs standardization to ensure interoperability between different manufacturers, security, and ethical management of collected data. Actually, standards like ISO 26262 [120], ISO 21448 [121], and General Data Protection Regulation (GDPR) [122] ensure that electronic systems and privacy aspects are safe and reliable.

## 7. Conclusions

Automotive security systems and traffic monitoring technologies are essential to keep cars safe, driving easier, and traffic flowing more smoothly by using advanced surveillance and data-driven solutions. To gain deeper insights into the current systems and future applications of AI tools in this field, this paper serves as a supplementary effort to the knowledge of AI-driven solutions from both academic and industry perspectives. Following a rigorous selection of scientific literature using the PRISMA methodology, this paper provides a comprehensive overview of IoV solutions for driver state monitoring and detection, vehicle monitoring, and traffic and road management. For each discussed topic, a comparative analysis and related discussion are reported to highlight the strengths and weaknesses of the analyzed solutions. Finally, the challenges and future perspectives are presented, bringing out the main requirements and insights for the development of the next generation of automotive monitoring systems.

## Figures and Tables

**Table 1:** Main characteristics of driver health monitoring systems in the literature.

Reference	Application	Acquisition Device	Detected Parameters	Intrusiveness	Alarm Systems	AI Algorithm	Accuracy
K. Kaur et al. [11]	Health monitoring	Six unspecified wearable sensors	ECG, BP, BT, HR, RR, BAC	High	Yes	RF, EC, DT	99.86% (RF) 99.83% (EC) 99.70% (DT)
A. Affanni et al. [12]	Well-being monitoring	Smart necklace	HR, blood oxygen saturation (SpO_2_)	Medium	No	“Mountaineer algorithm”	N.A.
Z. Gong et al. [13]	Heart rate monitoring (rPPG)	Camera	HR (rPPG)	Low	No	LAB algorithm by SenseTime	Pearson correlation coefficients: 0.943 (night) 0.906 (day)
Y. Jiao et al. [34]	Fatigue detection	Accelerometers	HVR, EDA	Low	No	LGBM, RF	88.70% (LGBM) 85.60% (RF)
L. Leicht et al. [36]	Health monitoring	MI coil, PPG sensor in seatbelt, camera, capacitive ECG sensor into seat	HR, RR	Medium	No	Pan–Tompkins algorithm, FaceLAB 5	N.A.
A.E. Campos-Ferreira et al. [37]	Health monitoring	Biometric wristband	HR, skin temperature	Medium	No	Principal Component Analysis (PCA)	N.A.
T. Škorić [38]	Health monitoring	Direct contact electrodes, capacitive electrodes in cushion and car seat	ECG, cECG	High (direct contact), Low (indirect contact)	No	KNN, SVM, ANN	100 % 96.67 % 98.08 % (^1^)

(^1^) The accuracy values refer to the authors’ three ECG signal detection methods, direct contact (100%), and indirect contact (96.67% in the case of sensors embedded into cushion and 98.08% in the case of sensors embedded into car seat); N.A.: Not Available.

**Table 2:** Main characteristics of driver drowsiness detection systems in the literature.

Reference	Application	Acquisition Device	Detected Parameters	Intrusiveness	Alarm Systems	AI Algorithm	Accuracy
S. Ebrahimian et al. [39]	Drowsiness detection	eWave32D, thermal camera	HR, RRV	Medium	No	CNN-LSTM	91% (^1^) 67% (^2^)
J. Alguindigue et al. [40]	Drowsiness detection	Smart band, smart glasses	HRV, EDA, PERCLOS	Medium	No	SNN, 1D-CNN, CRNN	98.28% (^3^) 96.32% (^4^)
A.S. BaHammam et al. [41]	Drowsiness detection (JDS)	Optalert glasses	Eyelid velocity, blink duration.	Low	No	Optalert^TM^ Proprietary pattern recognition algorithm	N.A. (*)
N. Sukumar et al. [14]	Detection of stress and drowsiness levels	Accelerometer, capacitive sensor	ECG, head movement, IDT	Medium	No	CWT-SST	91.82% (^5^) 97.30% (^6^)
A. Amidei et al. [42]	Drowsiness detection	Smart band	Skin conductance	Medium	No	RF	84.1%
S. Díaz-Santos et al. [43]	Drowsiness detection	Camera	Facial recognition	Low	No	OpenCV CNN	97.5%
M. A. Khan et al. [44]	Drowsiness detection	Camera	Facial features	Low	No	Unspecified	96%
F. Safarov et al. [15]	Drowsiness detection	Camera	Blink rate	Low	Yes	Computer vision algorithm	95.8% (^7^) 97% (^8^)
T. Kundinger et al. [45]	Drowsiness detection	Smart band	ECG	Medium	No	KNN	92.13%
D. A. Vyas et al. [46]	Detection of stress and aggression level of the driver	Mobility sensors, sensors for biophysical signals	EEG, PPG, ECG, SpO_2_	High	Yes	KNN, RF	86.05% (KNN) 88.24% (RF)

(^1^) Three-level drowsiness classification; (^2^) five-level drowsiness classification; (^3^) HRV detection; (^4^) EDA detection; (^5^) stress recognition; (^6^) drowsiness detection; (^7^) drowsy eye detection; (^8^) open eye detection; (*) N.A.: Not Available.

**Table 3:** Main characteristics of drunkenness detection methods in the literature.

Reference	Application	Acquisition Device	Detected Parameters	Intrusiveness	Alarm Systems	Algorithm	Performance
T.S. Kumar et al. [47]	Drunkenness detection	MQ-3 sensor, GPS module	BAC	Low	Yes	Threshold-based algorithm	85–94%
J. Liu et al. [48]	Drunkenness detection	MQ-3 sensor	BAC	Low	Yes	Data fusion and threshold-based algorithm	N.A. (*)
M. Swarna et al. [49]	Drunkenness detection	MQ-3 sensor, GPD module	BAC	Low	Yes	IIoTDSM	Efficiency: 98.70%
F. Wang et al. [50]	Driver and passenger drunkenness detection	Array of 21 gas sensors (^1^)	BAC	Low	Yes	KNN, SVM, RF	99.44% (I Step) 100% (II Step)
J. Akanni et al. [51]	Drunkenness detection	MQ-3 sensor	BAC	Low	Yes	Threshold-based algorithm	N.A. (*)
Y. Cho et al. [52]	Drunkenness detection	Spectroscopic module	BAC	Low	Yes	Absorbance vs alcohol characteristic	0.9908 (^2^) 0.9938 (^3^)

(^1^) Reported in Table 1 of Ref. [50]; (^2^) R-squared value for first-order equation; (^3^) R-squared value for second-order equation;.(*) N.A.: Not Available.

**Table 4:** Main features of vehicle diagnostic systems reported in Section 3.1.

Reference	Application	Sensors	Vehicular Parameters	Driving Parameters	Vehicle OBD-II Interaction	Detection Model	Performance
X. G. Yang et al. [18]	Fault diagnosis	Unspecified (^1^)	Unspecified (^1^)	Unspecified (^1^)	Yes	AUTOSAR	Detection rate: 98.70%, Response time: 0.0217 s
J. Kim et al. [56]	Fault diagnosis	6-D IMU sensor	Tire longitudinal and lateral force	Speed, heading	No	CNN	Accuracy: 99.50 %
Kannan et al. [57]	Fault diagnosis in a PdM context	On-board sensors (OBDII)	Engine data, transmission data, control unit	N.A.	Yes	LSTM	Accuracy: 99.62% Sensitivity: 100% Specificity: 100%
Min et al. [58]	Framework on sensor self-diagnosis	Unspecified (^2^)	Unspecified (^2^)	Unspecified (^2^)	No	DSAE	AUC_ROC: 0.8516 F1-score: 0.6701
J.P. Shermila et al. [59]	Automotive black box system	DHT 111, MQ-2, SW-18010p	Engine temperature	Speed, acceleration	Yes	Proprietary algorithm	Accuracy: 98% Recall: 90% Specificity: 85% Precision: 75%

(^1^) Ref. [18] studies a diagnostic software without explicitly referring to specific types of sensors; (^2^) the model proposed in [58] is an algorithm for identifying and isolating faulty sensors, and as such, it does not refer to specific types of sensors.

**Table 5:** Main features of innovative systems for detecting vehicle instability conditions described in Section 3.2.

Reference	Application	Sensors	Detection Model	Performance
A. Fichtinger et al. [60]	Aquaplaning detection	MARWIS (^1^)	Threshold-based algorithm	1.5 s detection time
J. Hu et al. [22]	Slippery road conditions detection	Ground speed, wheel speed, ground acceleration, and wheel acceleration	LSTM	SR: 100% (dry road), SR: 99.06% (snowy road), SR: 98.02% (icy road)
H. Lee et al. [62]	Black ice detection	Camera	CNN	Accuracy: 96%
S. Bhadraray et al. [63]	Detection of potholes on the road surface	Turtlebot3 Burger robot with RGB camera	YOLOv4-Tiny	N.A. (*)
G. Raja et al. [64]	Smart pothole avoidance system (SPAS)	Camera	DDPG and HRM-SG	Accuracy: 94.2%

(^1^) Mobile Advanced Road Weather Information Sensor; (*) N.A.: Not Available.

**Table 6:** Main features of systems for driver style and behavior monitoring reported in Section 3.3.

Reference	Application	Sensors	Vehicular Parameters	Driving Parameters	Vehicle OBD-II Interaction	Detection Model	Performance
R. Nouh et al. [66]	Driver risk classification	Speed sensor and accelerometer	-	Speed, acceleration	No	Collaborative Filtering (CF)	MAE: 88.41% MAPE: 90.18% MSE: 95.12%
K. Mohammed et al. [9]	Driving style tracking	GPS, on-board sensors (OBD-II)	Engine speed, coolant temperature, geolocation coordinates	Speed	Yes	-	N.A. (*)
Y. Shichkina et al. [67]	Driving style recognition	Stereo sensor, camera, on-board sensors (OBD-II)	Mass airflow, engine load, intake manifold pressure	Speed, throttle position, acceleration	Yes	Detectron2	N.A. (*)
R. Chhabra et al. [68]	Driver behavior classification	Accelerometer, gyroscope	-	Acceleration and angular speed	No	CNN-LSTM, CNN-Bi-LSTM	CNN-Bi-LSTM: 87%
M. Malik et al. [69]	Driving behavior	On-board sensors (OBD-II)	Engine RPM, engine load, coolant temperature	Speed and throttle position	Yes	ANN	Accuracy: 98.68%
E. Lattanzi et al. [70]	Driving behavior	On-board sensors (OBD-II)	Engine speed, engine load	Speed, brake pedal pressure, throttle position, steering wheel angle	Yes	SVM FFNN	Accuracy: 95%
B.T. Dong et al. [71]	Fatigue detection, distracted behavior	S3FD	-	Facial features	No	RF CNN	Accuracy: 91% for fatigue detection, 97.5% for distracted behavior

(*) N.A.: Not Available.

**Table 7:** Main features of the tire monitoring models reported in Section 3.4.

Reference	Application	Sensors	Vehicular Parameters	Driving Parameters	Vehicle OBD-II Interaction	Detection Model	Performance
A. Vasantharaj et al. [55]	TPMS	WSS, pressure sensors	Wheel speed	-	Yes	DSAE-ANN	Accuracy: 98.69%
Z. Màrton et al. [21]	iTPMS	WSS	Gear shift, sharp turn detection	Acceleration, deceleration	No	CNN, HWFT-64	Accuracy: 97.32% (HWFT-64)
T. Shan et al. [72]	iTPMS	WSS, on-board hardware	Vertical and circumferential vibrations, wheel speed	-	No	Threshold-based model	N.A. (*)
B. Huo et al. [73]	TPMS	Magnetoelectric WSS	Wheel speed	-	No	Threshold-based model	Accuracy: 79.3% (Radius); 95.6% (Frequency); 94.5% (Composite)
H. Yu et al. [75]	Tire wear detection	Magnetic angular velocity sensor, CAN (^1^)	Engine torque, engine speed, yaw rate, four-wheel angular velocity	Acceleration, brake	No	Transformer model	Accuracy: 97.77%

(^1^) Controller Area Network (CAN); (*) N.A.: Not Available.

**Table 8:** Main features of the emissions monitoring system reported in Section 3.5.

Reference	Application	On-Board Sensors	Off-Board Sensors	Vehicular Parameters	Driving Parameters	Environmental and Other Parameters	Vehicle OBD-II Interaction	Detection Model	Performance
N. Anusha et al. [76]	Monitoring and analysis of polluting emissions	Emissions sensors (particulate, NO_x_, CO_2_), GPS, IoT devices, OBD-II	Lidar, cameras, air quality sensors, weather stations	Vehicle type, engine size, emissions level	Speed	Temperature, weather conditions, traffic density, road type	Yes	NN	N.A. (*)
J. Sao et al. [77]	Emissions prediction of diesel vehicles	PEMS (^1^)	Not considered	Engine torque, speed, coolant temperature fuel/air ratio, intake air mass flow	Speed	Not considered	Yes	ANN	N.A.
P. Andrade et al. [78]	CO_2_ emissions estimate	Engine Control Unit (EUC) sensors	Not considered	RPM, intake air temperature, mass air flow	Speed	Not considered	Yes	TEDA-based algorithm	Volumetric efficiency: 80%

(^1^) Portable Emission Measurement System (PEMS); (*) N.A. Not Available.

**Table 9:** Main features of traffic management systems reported in Section 4.1.

Reference	Application	IoT Sensors and Technologies	Communication Technology	Entities Involved in Communication	Monitoring Algorithm	Performance
U.K. Lilhore et al. [25]	Traffic management	Cameras, RFID tags, GPS module, WSNs (^1^)	IP-based internet 3G/4G, LTE, Wi-Fi, NFC, Bluetooth, ZigBee	Vehicle, roadside infrastructure, and events	DBSCAN clustering method	N.A. (*)
M. Saleem et al. [80]	Traffic management	IoV sensors	Internet (IoV)	Vehicle, roadside infrastructure	FITCCS-VN	Accuracy: 95% Miss rate: 5%
V. Mandal et al. [82]	Traffic monitoring	Cameras	N.A. (*)	Traffic monitoring centers	YOLO	Accuracy: 93.7% F1-score: 0.8333 RMSE: 154.7741 S3: 0.4034
M. Humayun et al. [83]	Traffic management	Cameras, magnetic sensors	5G/Wi-Fi	Vehicle, roadside infrastructure, mobile user.	Threshold-based method	N.A. (*)
A.S. Putra et al. [84]	Traffic management	Motion, ultrasonic, PIR, and speed sensor	Internet network, cellular wireless technology	Vehicle, mobile user	-	N.A. (*)
M. Sarrab et al. [85]	Traffic management	Magnetic sensors, NodeMCU	Wi-Fi	Roadside infrastructure	Threshold-based method	Functionality evaluation: 80%
R. Barbosa et al. [86]	Traffic monitoring and vehicle identification	Image sensor	5G	Roadside infrastructure	Light-SpaN	Performance: 99.9%
M.Q. Kheder et al. [87]	Traffic monitoring systems with VCC	IR, GPS, ultrasonic sensor, cameras	Wi-Fi, Bluetooth, cellular networks	Vehicle, commuters, Google’s firebase	LeNet-5 Inception-V3	Performance: 99.7% Performance: 98.6%
S. Dhingra et al. [88]	Urban traffic monitoring and light management	HC-SR04 Ultrasonic Sensor	Wi-Fi, BLE	Roadside infrastructure	Cloud computing	N.A. (*)
N.A. Khan et al. [89]	Traffic surveillance system based on UAVs	Cameras, GPS module	5G	UAV, driver, base station	Threshold-based method	N.A. (*)
S. Singh et al. [90]	Navigation reference spatial data	Open street map, GPS module	Unspecified	Roadside infrastructure	Bayesian classifier	Accuracy: 98% Performance: 87%

(^1^) Wireless Sensor Nodes (WSNs); (*) N.A. Not Available.

**Table 10:** Main features of environmental and road condition monitoring systems reported in Section 4.2.

Reference	Application	IoT Sensors and Technologies	Communication Technology	Entities Involved in Communication	Monitoring Algorithm	Performance
M. Åstrand et al. [91]	Road condition monitoring	Inertial and wheel speed sensors	Wi-Fi	WiFi access points	Kalman filter-based model	N.A. (*)
Z. Ye et al. [92]	Vehicle type, speed, and weight; pavement health status and environment monitoring	Vibration, temperature, humidity, and wind sensors, cameras	5G	Roadside infrastructure (5G gateway), remote server	Threshold-based model	±1 cm localization accuracy, 100 % survival rate
M.E.S. Abdelmalak et al. [93]	Pothole detection on asphalted roads	Cameras, IMU, GPS module	LoRa	Unmanned ground vehicles (AGV) and ground station	AlexNet	Accuracy: 92.15% Sensitivity: 91.38% F1-score: 96.52%
Z. Ye et al. [94]	Road monitoring system	WSNs (acceleration sensing nodes and gateway)	LoraWand, UDP, HTTP communication protocol	Front-end devices and back-end platform	Time features processing	Vehicle speed and whee-lbase error < 2%
G. Gaspar et al. [95]	Road infrastructure management	Temperature sensors	Generic wireless transmission technology	DBAR logger, server, database	N.A.	N.A. (*)
M. Hajder et al. [96]	Road maintenance	Temperature sensors, humidity sensors	3G and 4G/LTE communication	Unspecified	LSTM	Accuracy: 89%
Z. Chen et al. [97]	Road icing detection and prediction	Ice detection sensor, DS18B20 temperature sensor	Wireless communication based on Semtech SX1278	Roadside infrastructure	Position encoding multi-head attention mechanism, GAN-based classification	Precision: 0.743 Recall: 0.811 F1-score: 0.767
J. Kotus et al. [98]	Wet road detection	Acoustic vector sensor	Unspecified	Roadside infrastructure	Threshold-based model, spectral analysis	Accuracy: 89%

(*) N.A. Not Available.

**Table 11:** Comparison of commercial DMSs illustrated in Section 5.1.

Commercial DMS	Detection Capabilities	Sensing Devices	Body and Vehicle Parts Monitored	Installation Area	Detection Algorithm
Smart Eye Pro 12 by SmartEye [99]	Driver identification, distraction, drowsiness, dangerous behaviors, body posture, facial expressions	1 MP cameras	Body posture, head and eye position, eye gaze and blinking, pupil diameter	Front of the cockpit	AI-based emotion
Cabin Sensing Solution by Continental Engineering [100]	Facial expressions and recognition, driver distraction, drowsiness, head and forehead temperature, RR, HR	1 MP camera, radar, thermal camera	Head and eye position, seatbelt, seat occupancy	Front of the cockpit	Proprietary software
Driver and Occupant Monitoring System by Magna International Inc. [101]	Driver identification, distraction, drowsiness, fatigue, facial expressions, ethanol environmental concentration	1.7 MP camera, IR movement and ethanol sensors	Head and eye position, seatbelt, seat occupancy	Rear-view mirror and steering block	Proprietary software
Driver Fatigue Monitoring System by Speedir Inc. [102]	Driver distraction, drowsiness, facial expressions	Night vision camera, IR sensors	Head and eye position, eye gaze and blinking, pupil diameter	Front of the cockpit	Pre-trained AI software

**Table 12:** Comparison of commercial IVMSs illustrated in Section 5.2.

Commercial IVMS	Applications	Cameras and Other Devices	Sensors	Monitored Vehicle Parameters	Connectivity Technologies and Interfaces	Software
Trakm8 system [104,105,106]	Driver ID and behavior, vehicle health, ADAS functionalities	4G camera, dash cam, 4G mobile digital video recorder, driver ID and feedback device	G-Sensor and radar	Washer fluid level, ABS, traction control, DPF, AdBlue levels, tire pressure	BLE 4.2, GPS, Wi-Fi, 4G technology, CAN 1 and Tacho CAN 2 interfaces	ConnectedCare, insight telematics, driver ID authentication
TrackoBit by InsightGeeks Solutions Pvt. Ltd. [107]	Driver behavior, ADAS functionalities, geospatial tracking, route planning and management	AI-powered DMS and camera	Radar	-	GPS, 4G technology	GPS tracking software, video telematics, route planning and management software
IVMS by Forvia [108,109,110,111,112,113,114]	By-wire technology and ADAS functionalities	eMirrors and surround-view cameras	Brake and steering torque sensors, road condition piezoelectric sensor, rain light and parking sensors, 77 GHz radar	-	OBD port, GPS, 4G technology	eMirror UX Safe engine

## Data Availability

The data are available upon request.
